# Protective Effects of Lotus Seedpod Extract on Hepatic Lipid and Glucose Metabolism via AMPK-Associated Mechanisms in a Mouse Model of Metabolic Syndrome and Oleic Acid-Induced HepG2 Cells

**DOI:** 10.3390/antiox14050595

**Published:** 2025-05-16

**Authors:** Hui-Hsuan Lin, Pei-Rong Yu, Chiao-Yun Tseng, Ming-Shih Lee, Jing-Hsien Chen

**Affiliations:** 1 Department of Medical Laboratory and Biotechnology, Chung Shan Medical University, Taichung City 40201, Taiwan; linhh@csmu.edu.tw; 2 Clinical Laboratory, Chung Shan Medical University Hospital, Taichung City 40201, Taiwan; 3 Department of Nutrition, Chung Shan Medical University, Taichung City 40201, Taiwan; 1046004@live.csmu.edu.tw (P.-R.Y.); 1146002@live.csmu.edu.tw (C.-Y.T.)

**Keywords:** metabolic syndrome, lotus seedpod extract, lipid dysmetabolism, insulin resistance

## Abstract

Metabolic syndrome (MetS) poses considerable toxicological risks due to its association with an increased likelihood of metabolic dysfunction-associated steatotic liver disease (MASLD), and is characterized by hypertension, hyperglycemia, dyslipidemia, and obesity. This study aimed to investigate the therapeutic potential of flavonoid-rich lotus seedpod extract (LSE) in alleviating MetS and MASLD-related hepatic disturbances. In vivo, mice subjected to a high-fat diet (HFD) and streptozotocin (STZ) injection were supplemented with LSE or simvastatin for 6 weeks. Obesity indicators included body weight and epididymal fat, while insulin resistance was measured by fasting serum glucose, serum insulin, homeostasis model assessment–insulin resistance index (HOMA-IR), and oral glucose tolerance (OGTT). Also, the levels of serum lipid profiles and blood pressure were evaluated. Adipokines, proinflammatory cytokines, liver fat droplets, and peri-portal fibrosis were analyzed to clarify the mechanism of MetS. LSE significantly reduced the HFD/STZ-induced MetS markers better than simvastatin, as demonstrated by hypoglycemic, hypolipidemic, antioxidant, and anti-inflammatory effects. In vitro, LSE improved oleic acid (OA)-triggered phenotypes of MASLD in hepatocyte HepG2 cells by reducing lipid accumulation and enhancing cell viability. This effect might be mediated through proteins involved in lipogenesis that are downregulated by adenosine monophosphate-activated protein kinase (AMPK). In addition, LSE reduced reactive oxygen species (ROS) generation and glycogen levels, as demonstrated by enhancing insulin signaling involving reducing insulin receptor substrate-1 (IRS-1) Ser307 phosphorylation and increasing glycogen synthase kinase 3 beta (GSK3β) and protein kinase B (PKB) expression. These benefits were dependent on AMPK activation, as confirmed by the AMPK inhibitor compound C. These results indicate that LSE exhibits protective effects against MetS-caused toxicological disturbances in hepatic carbohydrate and lipid metabolism, potentially contributing to its efficacy in preventing MASLD or MetS.

## 1. Introduction

Metabolic syndrome (MetS) is a group of metabolic dysregulations characterized by hyperglycemia, abdominal obesity, atherogenic dyslipidemia, and systemic hypertension, all of which collectively increase the risk of type 2 diabetes mellitus (T2DM), cardiovascular disease, and liver complications. The underlying pathophysiology of MetS has been linked to several key mechanisms, including chronic inflammation, insulin resistance, and neurohormonal activation, particularly involving the overactivation of the renin–angiotensin–aldosterone system (RAAS) and the sympathetic nervous system, which further exacerbate metabolic and cardiovascular dysfunction [1]. To recapitulate the pathogenesis of T2DM and its associated metabolic disorders in experimental models, rodents are often fed a high-fat diet (HFD) to induce insulin resistance and obesity, followed by a low-dose injection of streptozotocin (STZ) to mimic pancreatic β-cell dysfunction. This combined HFD/STZ protocol closely reflects the human condition of T2DM, characterized by hyperglycemia, dyslipidemia, and impaired glucose tolerance [2]. Importantly, the HFD/STZ model also induces liver steatosis, inflammation, and fibrotic changes that resemble metabolic dysfunction-associated steatotic liver disease (MASLD). T2DM and MASLD are interrelated conditions, with MASLD predisposing individuals to T2DM, while existing T2DM heightens the risk of MASLD onset and accelerates its progression to fibrosis. MASLD refers to a spectrum of hepatic disorders characterized by excessive lipid accumulation resulting from metabolic dysfunction, such as in metabolic dysfunction-associated steatotic liver (MASL), and is defined as occurring in the absence of significant alcohol consumption [3,4]. In a particular cohort of individuals with MASL, the condition may develop into a severe hepatic illness known as metabolic dysfunction-associated steatohepatitis (MASH). MASH is defined by hepatocellular injury, inflammation, and the formation of scar tissue, leading to fibrogenesis [5].

Among the multiple-hit pathogenesis of MASLD, insulin resistance is vital in the development of steatosis and MASH. Glycogen synthase kinase 3β (GSK3β) is recognized for its role in inhibiting insulin function through the phosphorylation and subsequent inactivation of insulin receptor substrate-1 (IRS-1) [6]. Upon insulin stimulation, the linear activation of the insulin receptor, insulin receptor substrate-1, and protein kinase B (IR/IRS-1/Akt) inactivates GSK3, leading to the activation of glycogen synthesis and the dephosphorylation of glycogen synthase (GS) [7]. Dysfunctions in insulin signaling pathways have been associated with abnormal phosphorylation of serine residues in adaptor IRS-1 and IRS-2 proteins [8]. Additionally, insulin resistance leads to adipose tissue dysfunction, resulting in increased generation and secretion of inflammatory cytokines and adipokines [9]. Steatosis contributes to elevated levels of the transcription factor–nuclear factor kappa B (NF-κB) and stimulates synthesis of proinflammatory factors such as interleukin (IL)-6 and 1β, and tumor necrosis factor-alpha (TNF-α). These cytokines function in recruiting Kupffer cells, which are macrophages located in the liver, to initiate inflammation in MASH [10].

Studies have indicated that insulin resistance not only impairs the suppression of adipose tissue lipolysis but also promotes higher hepatic de novo lipogenesis (DNL), resulting in an elevated flux of FA to the liver [11]. When FA overloads the liver’s physiological adaptive systems, esterification may increase triglyceride (TG) production, ROS generation, lipotoxicity, and hepatocellular damage [12]. Moreover, the rate-limiting enzyme, 3-hydroxy-3-methylglutaryl coenzyme A (HMG-CoA) reductase (HMGCR), converts HMG-CoA into mevalonate, determining de novo cholesterol (CHOL) synthesis. The sterol regulatory element-binding proteins (SREBPs) modulate the transcriptional expression of the HMGCR gene [13]. In MASLD patients, lipogenesis rates are three times higher than in the general population, with persistent activation of SREBP1c boosting lipogenic activity, hence accelerating hepatic steatosis [14]. In addition, free CHOL increases in MASLD and correlates with SREBP-2 induction [15].

*Nelumbo nucifera* is often known as the lotus and is an aquatic plant widely distributed in Asia, Australia, and parts of the Americas. The lotus is highly valued for its beautiful, fragrant flowers; large, round leaves; and ecological importance. Almost all portions of this plant have been utilized as herb medicines and functional food [16]. The pharmaceutical activities of lotus are commonly attributed to its active components, which encompass a variety of phytochemicals such as flavonoids, alkaloids, polysaccharides, essential oils, triterpenoids, and steroids. Lotus seedpod was generally disregarded and abandoned, with its limited use primarily being as a traditional Chinese herbal remedy for enhancing blood circulation and eliminating blood stasis [17]. Recent pharmacological research has shown that procyanidins from lotus seedpods possess a diverse array of biological activities beneficial to human health, which include radical-scavenging activity, memory-protective effects, and anti-bacterial properties [18]. For the standardization of lotus seedpod aqueous extract (LSE), the component identification approaches have been performed, and it has been detected that quercetin-3-glucuronide [Q3G; 122.44 ± 2.24 mg/100 g dried weight (DW)] was shown in the highest content in LSE, followed by isorhamnetin-3-glucuronide (30.27 ± 3.46 mg/100 g DW) being identified, confirming that flavonoids were rich in LSE (Table S1). A prior study has revealed the LSE improves diabetes mellitus symptoms and demonstrates a protective effect on pancreatic beta cells against oxidative damage [19]. Furthermore, LSE exhibits hepatoprotective effects against lipopolysaccharide-induced hepatic inflammation [20].

Therefore, this study aimed to investigate the hepatoprotective effects of a flavonoid-rich LSE in the context of metabolic syndrome and MASLD, using both in vivo and in vitro models. Distinct from prior research primarily centered on glycemic regulation [19], this study specifically evaluates the effects of LSE on hepatic steatosis, inflammation, and fibrosis—hallmark pathological features of MASLD progression. Mechanistically, the modulation of insulin signaling pathways, lipid metabolism, and oxidative stress were explored. Additionally, to substantiate the translational relevance of our findings, a complementary in vitro model employing human hepatocellular HepG2 cells was utilized to elucidate the involvement of adenosine monophosphate-activated protein kinase (AMPK) signaling in LSE-mediated hepatic protection. To our knowledge, this is the first comprehensive investigation integrating both systemic and cellular models to characterize the therapeutic potential and mechanistic basis of LSE in MASLD. These findings may offer novel insights into the development of LSE as a candidate nutraceutical for the management of MASLD and associated metabolic dysregulations.

## 2. Materials and Methods

### 2.1. Extraction of Lotus Seedpod

The unprocessed lotus seedpods from Baihe District (Tainan, Taiwan), which originate to the *Nelumbo nucifera* Gaertn plant (cultivar: Sheklian). The 100 g of raw lotus seedpods were boiled in 4 L of water heated to 95 °C for a duration of 2 h. The aqueous extract was filtered and subjected to Heto PowerDry PL3000 Freeze Dryer (Thermo Fisher Scientific, Waltham, MA, USA), resulting in the production of a powder. This process yielded an aqueous portion of lotus seedpod extract (LSE) with a rate of roughly 27%. The resulting extract powder was then stored at −80 °C until it was ready for use [19,20].

### 2.2. In Vivo Experiments

The Institutional Animal Care and Use Committee of the Chung Shan Medical University animal care committee approved (IACUC approval number: 1404) the housing of five-week-old male BALB/c ByJNarl mice, which were purchased from the National Laboratory Animal Center (Taipei, Taiwan). After one week of adaption, the mice were randomly allocated into the following groups: (i) control (normal diet), (ii) HFD/STZ (high-fat diet coupled with STZ injection) (iii) HFD/STZ + 1% LSE (1% *w*/*w*, mixed in feed), (iv) HFD/STZ + 2% LSE (2% *w*/*w*, mixed in feed), and (v) HFD/STZ + simvastatin (clinical medication). To establish a MetS/MASLD model, mice were fed a combination of HFD and low-dose STZ, as previously described with modifications [2]. This combination was selected to better recapitulate the natural progression of human T2DM, from insulin resistance to β-cell dysfunction and persistent hyperglycemia, thereby more accurately reflecting the complex metabolic disturbances observed in human MetS and T2DM [21]. In addition, the combination model also induces hepatic steatosis, inflammation, and fibrotic alterations that closely resemble the pathophysiological features of MASLD, making it a suitable model for investigating the metabolic and hepatic complications associated with MetS and MASLD [22]. Group II-V mice were fed an HFD formula (#58Y1; TestDiet, Richmond, IN, USA), containing 61.9% fat, 17.8% protein, and 20.3% carbohydrate as a percentage of total kilocalories, for 12 weeks. Following a 5-week HFD treatment, mice in groups II-V were administered STZ (intraperitoneal injection, 40 mg/kg body weight) for five consecutive days. The fasting blood glucose values were monitored after five days of STZ administration to establish the diabetic model [23]. Based on previous study [19], the experimental design was modified as follows: groups III and IV were subsequently fed diets supplemented with 1% and 2% LSE, respectively, for 6 weeks. In addition, group V was treated with simvastatin (30 mg/kg body weight/day), a statin drug, via oral gavage (p.o.) for 6 weeks as a clinical medicine control. Mice in group I received a normal diet (#5010; LabDiet, St. Louis, MO, USA) and intraperitoneal injections of vehicle (normal saline) following the experiment period, serving as the control group. After the animal experiments, the liver sections were removed expeditiously and stored under a temperature of −80 °C. The thiobarbituric acid-reactive substances (TBARS) test, adipokines, proinflammatory cytokines, antioxidant enzyme assessment, and Western blotting were all performed on the liver tissue homogenate.

### 2.3. Assessment of Metabolic Parameters and Insulin Resistance

The mice were observed weekly for body weight, and the values were recorded throughout the treatment period. On the day before sacrifice, an oral glucose tolerance test (OGTT) was conducted. A 2 g/kg oral glucose solution was given to the mice. Tail vein blood collection was performed from each group at 30, 60, 90, and 120 min intervals to measure glucose levels in mice. Immediately following the sacrifice, epididymal fat was weighted and recorded. In addition, serum insulin levels were measured using a Mouse Insulin ELISA kit (Mercodia, Uppsala, Sweden) according to the manufacturer’s instructions. Briefly, serum samples and standards were added to a 96-well plate pre-coated with anti-insulin antibodies, followed by incubation with enzyme-conjugated secondary antibodies. After washing, a chromogenic substrate was added, and absorbance was measured at 450 nm using a microplate reader. Insulin concentrations were calculated based on a standard curve. Furthermore, the homeostasis model assessment-insulin resistance index (HOMA-IR) was calculated using glucose and insulin values: HOMA-IR. The HOMA-IR calculation is determined by multiplying the fasting insulin (μU/mL) by the fasting glucose (mmol/L) and then dividing the result by 22.5 [24].

### 2.4. Evaluation of Hepatic Injury and Fibrosis

For histopathological examination, the liver tissue slices fixed in paraffin were stained with hematoxylin and eosin (H&E) and Masson’s trichrome, in accordance with the methods defined previously [25]. For H&E staining, the NAFLD activity score (NAS) was used to semi-quantitatively assess the severity of hepatic lesions, which reflect activity of MASLD/MASH [26]. The scoring system includes steatosis (0–3), lobular inflammation (0–3), and hepatocellular ballooning (0–2), with a total score ranging from 0 to 8. Each feature was assessed in randomly selected fields at 200× magnification by three independent observers blinded to the experimental groups. The NAS is the sum of these scores, with values ≥ 5 being correlated with a diagnosis of NASH in humans [27]. For Masson’s staining, the Masson’s-positive area, representing collagen deposition, was quantified using ImageJ software (version 1.51k, NIH, Bethesda, MD, USA).

### 2.5. Assessment of Serum Lipid Profile, Glucose, and Hepatorenal Function Indicators

The blood samples from mice were collected and centrifuged to isolate the serum samples. The serum was subjected to analysis utilizing a biochemical analyzer (Hitachi 7020 chemistry analyzer, Hitachi Co., Ltd., Tokyo, Japan) to determine biochemical parameters as follows: the serum lipids, including triglyceride (TG), cholesterol (CHOL), low-density lipoprotein cholesterol (LDL-c), high-density lipoprotein cholesterol (HDL-c), as well as the blood glucose (Glc), and the liver/renal function markers such as glutamic oxaloacetic transaminase (GOT), glutamic pyruvic transaminase (GPT), and blood urea nitrogen (BUN).

### 2.6. Measurement of Blood Pressure

Systolic blood pressure (SBP), diastolic blood pressure (DBP), and mean blood pressure (MBP) were measured in conscious mice using a non-invasive tail cuff system (MK-2000, Muromachi Kikai Co., Tokyo, Japan). During the procedure, mice were placed in a quiet, dimly lit environment to minimize external stress and were kept on a warming pad to maintain body temperature and ensure adequate tail blood flow. Blood pressure was measured once per week throughout the experimental period. For each session, at least five consecutive readings were taken per mouse, and the average value was used for analysis.

### 2.7. Thiobarbituric Acid-Reactive Substances (TBARS) Test

The degree of lipid peroxidation in cell lysate or liver homogenate was determined using the TBARS assay as described previously [28]. The production of TBARS by reacting homogenate malondialdehyde (MDA) with thiobarbituric acid (TBA) in an acidic buffer was utilized to quantify lipid peroxidation. In addition, TBARS concentration was calculated by comparing its values to a standard curve of MDA equivalents produced through the 1,1,3,3-etramethoxypropane (Sigma-Aldrich, St. Louis, MO, USA), which was catalyzed hydrolysis by acid.

### 2.8. Adipokines Assay

The serum sample was analyzed for leptin and adiponectin levels using the murine leptin/adiponectin ELISA development kit from PeproTech, Inc. (Cranbury, NJ, USA). In a previous study [29], the leptin/adiponectin ratio has been suggested as a marker of adipose tissue dysfunction. This emerging biomarker correlates with insulin resistance more strongly than adiponectin or leptin alone, or even homeostasis model assessment (HOMA). Therefore, the leptin/adiponectin ratio has been proposed as a predictive marker for metabolic syndrome (MetS).

### 2.9. Proinflammatory Cytokines Levels Analysis

The instructions described in the datasheet were followed to assess the concentrations of IL-1β, IL-6, and TNF-α in the blood samples from each group using an enzyme-linked immunosorbent assay (ELISA) with the ELISA MAXTM Deluxe Sets (BioLegend, San Diego, CA, USA).

### 2.10. Antioxidant Enzymes Activity Assays

To assess the glutathione (GSH) level, as well as the activities of glutathione peroxidase (GPx) and superoxide dismutase (SOD) in liver tissue homogenate, antioxidant enzyme assay kits from Cayman Chemical Co. (Ann Arbor, MI, USA) were used following the guidelines outlined in the datasheet.

### 2.11. Western Blot (WB)

Protein concentrations were quantified using the Dual-Range BCA Protein Assay Kit (Energenesis Biomedical Co., Taipei, Taiwan). Protein samples were extracted from both mouse liver homogenates and HepG2 cell lysates. A total of 30 μg of protein from each sample was separated using 8–15% sodium dodecyl sulfate–polyacrylamide gel electrophoresis (SDS-PAGE), as previously described [30]. A standardized loading amount of 30 μg was applied across all experimental groups to ensure consistency and comparability of results. While protein loading can be optimized individually based on the expected expression levels, a fixed loading approach was adopted in this study to maintain uniform experimental conditions for semi-quantitative analysis. The separated proteins were then transferred onto nitrocellulose membranes (Millipore, Burlington, MA, USA), which were blocked with 5% fat-free milk in tris-buffered saline with 0.1% Tween-20 (TBST) for 1 h at 4 °C to minimize nonspecific binding. The membranes were incubated overnight at 4 °C with the indicated primary antibodies (listed in Table S2). The following day, membranes were washed three times (10 min each) with TBST at room temperature, followed by incubation with the diluted secondary antibodies—anti-mouse IgG (A9044) and anti-rabbit IgG (A0545), both from Sigma-Aldrich (St. Louis, MO, USA)—for 1 h at 4 °C with gentle shaking. After a final washing step, protein bands were visualized using enhanced chemiluminescence (ECL) reagents (Millipore, Burlington, MA, USA), and signal detection was performed using a Luminescent Image Analyzer (ImageQuant LAS-4000, GE Healthcare Bio-Sciences AB, Björkgatan, Uppsala, Sweden).

### 2.12. Cell Culture

The human hepatocyte HepG2 cells were purchased from the Bioresource Collection and Research Center (BCRC, Hsinchu, Taiwan). Minimal Essential Medium (MEM) supplemented with Earle’s balanced salt solution (EBSS) was administered to cultivate HepG2 cells. At 37 °C in a humidified environment with 5% carbon dioxide (CO_2_), the cell culture medium was supplemented with 2.2 g/L sodium bicarbonate (NaHCO_3_), 10% fetal bovine serum (FBS), 1% L-glutamine, 1% sodium pyruvate, 1% non-essential amino acids, and 1% penicillin-streptomycin. After seeding cells into 6-well plates, the cells were cultured until the cell density reached almost 60–70% confluence prior to treatment. HepG2 cells were stimulated with 0.6 mM oleic acid (OA; Sigma-Aldrich, St. Louis, MO, USA) to induce excessive fatty acid accumulation and were concurrently treated with or without the indicated concentrations of LSE (1 and 5 μg/mL) for 24 h. The OA stock solution was prepared based on a previously published method [30], with modifications. OA was dissolved in culture medium supplemented with 10 μL/mL bovine serum albumin (BSA; Sigma-Aldrich, St. Louis, MO, USA). The OA/BSA complex was then diluted with culture medium to the working concentration and sterile-filtered through a 0.22 μm membrane filter. A culture medium containing BSA alone was used as the control. All treatments were performed in triplicate (n = 3) and the experiments were independently repeated at least three times.

### 2.13. Trypan Blue Exclusion Test

To examine the effect of the experimental interventions on cell viability, the trypan blue dye exclusion experiment was conducted as previously described [20]. To evaluate the cytotoxicity of OA and LSE, different concentrations of OA (0, 0.1, 0.2, 0.5, 0.6, 0.8, and 1 mM) and LSE (0, 0.1, 0.5, 1, 5, 10, 50, and 100 µg/mL) were administered to HepG2 cells, respectively. Subsequently, the trypan blue dye was used to stain the cells, and the quantity of viable cells was counted to determine cell growth. Following the dose-screening test, HepG2 cells were exposed to 0.6 mM of OA with or without the specified doses of LSE (1 and 5 µg/mL).

### 2.14. Oil Red Staining

The treated cells were treated with 4% paraformaldehyde solution for around 30 min. Subsequently, the fixed cells were colored with oil red reagent (Sigma-Aldrich, St. Louis, MO, USA) for a fifteen-minute period. Micrographs were taken at a 100× magnification after the dyed cells were observed under a microscope. In addition, the oil red staining content in cells was determined by extracting the dye with isopropanol and then measuring the absorbance via spectroscopy at 490 nm.

### 2.15. Nile Red Staining

After the treatments, the cells were rinsed and subsequently treated with 4% paraformaldehyde for a duration of 30 min. The fixed cells were cultured with 1 μg/mL Nile red reagent (Sigma-Aldrich, St. Louis, MO, USA) for around 5 min and analyzed for fluorescence intensity by the Muse™ Cell Analyzer (Cytek Bioscience, Fremont, CA, USA) at 488 nm (excitation) and 550 nm (emission).

### 2.16. Reactive Oxygen Species (ROS) Level Assay

Dichlorofluorescein diacetate (DCFH-DA) from Enzo Life Sciences Inc. (Farmingdale, NY, USA) dyed the cells after the treatments. The intracellular ROS generation was determined by fluorescence intensity using the flow cytometry, and the data in each group were expressed in relation to the control, which served as 100%.

### 2.17. Glycogen Content Analysis

The treated cell lysates were tested for glycogen concentration using the EnzyChrom Glycogen Assay Kit from BioAssay Systems (Hayward, CA, USA). The procedure was performed as instructed in the datasheet.

### 2.18. Immunoprecipitation (IP) Assay

Protein A Mag Sepharose Xtra from Cytiva (Uppsala, Sweden) was added to 500 μg of total protein samples extracted from cell lysate. 5 μg primary antibody, IRS-1 (sc-560, Santa Cruz Biotechnology, Santa Cruz, CA, USA), was applied for immunoprecipitation on the protein samples. Next, the complexes, which had been precipitated, were examined using Western blotting with p-Tyr antibodies (9416S, Cell Signaling Technology, Danvers, MA, USA).

### 2.19. AMPK Inhibition Test

As an AMPK inhibitor, compound C (3 μM) was subjected to HepG2 cells before the treatments. After collecting the treated cells, 10 mg/mL propidium iodide (PI, Sigma-Aldrich, St. Louis, MO, USA) was used to dye the cells, and the cells were evaluated for cell viability by flow cytometry. All values were presented as percentages in relation to the control, which was set at 100%. Subsequently, oil red staining and Western blotting were carried out as described above.

### 2.20. Statistical Assay

All results were expressed as means ± standard deviation (SD), and all statistical analyses were performed using SAS Enterprise Guide 8.3 (SAS Institute Inc., Cary, NC, USA). Data normality was assessed using the Shapiro–Wilk test. For comparisons among multiple groups, one-way analysis of variance (ANOVA) was performed, followed by Tukey’s multiple comparison test as a post hoc analysis. A *p*-value of less than 0.05 was considered statistically significant.

## 3. Results

### 3.1. The Effects of LSE on MetS in Mice Following HFD/STZ Induction

**Figure 1:**
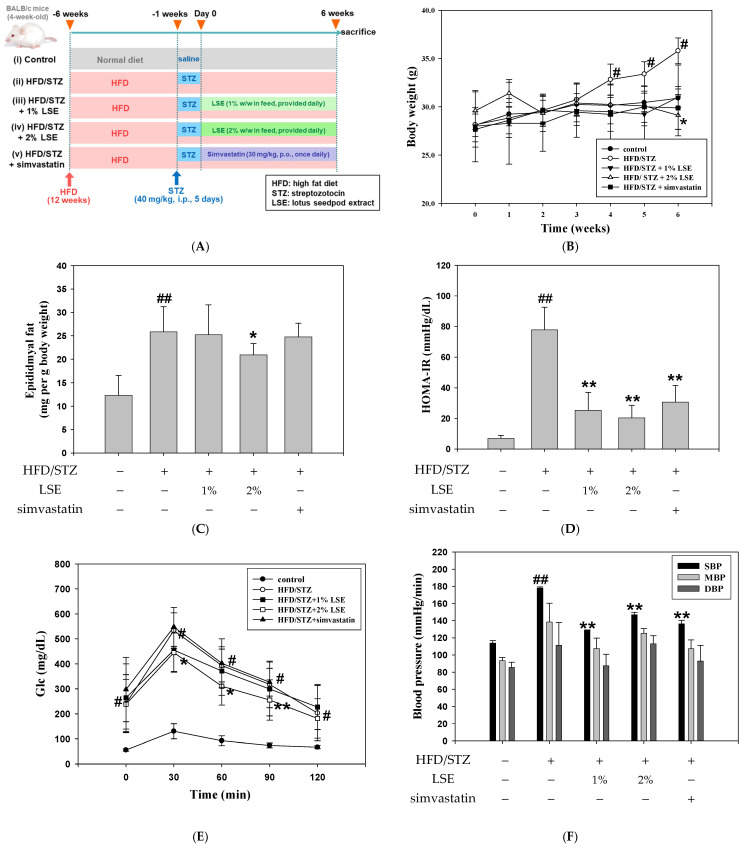
The effects of LSE on MetS in mice following HFD/STZ induction. (**A**) Schematic diagram of the experimental design. The MetS mice induced by HFD/STZ treatment (HFD for 12 weeks, followed by intraperitoneal injection of 40 mg/kg STZ for 5 consecutive days) were treated for 6 weeks with either LSE (1% or 2% *w*/*w* in feed), simvastatin (30 mg/kg, oral gavage, once daily), or vehicle (control group). (**B**) Body weight of mice was collected weekly for six weeks during the experiment. (**C**) The mass of epididymal fat was quantified as milligrams per gram of total body weight in several groups. (**D**) Fasting plasma glucose and insulin levels were used to compute HOMA-IR on week 6. Serum OGTT (**E**) and blood pressure (**F**) were also measured. The quantitative data were shown as mean ± SD (n ≥ 3), derived from at least three independent biological replicates. ^#^ *p* < 0.05, ^##^ *p* < 0.01 compared with the control group; * *p* < 0.05, ** *p* < 0.01 compared with the group of HFD/STZ. MetS: metabolic syndrome; HFD/STZ: high-fat diet/streptozotocin; LSE: lotus seedpod extract; HOMA-IR: homeostasis model assessment–insulin resistance index; OGTT: oral glucose tolerance test; SBP: systolic blood pressure; MBP: mean blood pressure; DBP: diastolic blood pressure).

The experimental procedure conducted in the in vivo experiment was illustrated in Figure 1A and followed the methodology described in a prior study [19]. In the animal experiment, simvastatin was included as a therapeutic control to benchmark the anti-lipogenic and anti-inflammatory effects of LSE, specifically targeting the hepatic metabolic disturbances associated with MASLD. Initially, the effect of LSE on HFD-/STZ-induced metabolic syndrome was examined by assessing physiological variables and metabolic disorder features, such as body weight (BW), epididymal fat, HOMA-IR, OGTT, and blood pressure (Figure 1B–F). The results of Figure 1B–F indicated a significant increase in BW, epididymal fat, HOMA-IR, OGTT, and blood pressure in the HFD/STZ group, indicating the various physical characteristics of metabolic syndromes. Figure 1B,C showed that the group treated with LSE exhibited the reductions in BW and epididymal fat relative to the HFD/STZ stimulation, particularly in the 2% LSE intervention. As illustrated in Figure 1D–F, not only the LSE group, but also the clinical medication simvastatin group, exhibited a significant reduction in HOMA-IR, OGTT, and systolic blood pressure (SBP) in comparison to the HFD/STZ group. In addition, the serum biochemical parameters were assessed to evaluate whether the MetS model worked, including serum lipids, liver functions, renal functions, and glucose. LSE treatment reversed the HFD/STZ-induced abnormalities in biochemical markers, including triglyceride (TG), cholesterol (CHOL), low-density lipoprotein cholesterol (LDL-c), glucose (Glc), glutamic oxaloacetic transaminase (GOT), glutamic pyruvic transaminase (GPT), and blood urea nitrogen (BUN). LSE improved metabolic syndrome features and serum biochemical parameters altered by HFD/STZ (Figure S1).

### 3.2. The Effects of LSE on Proinflammatory Cytokines in Mice Following HFD/STZ Induction

**Figure 2:**
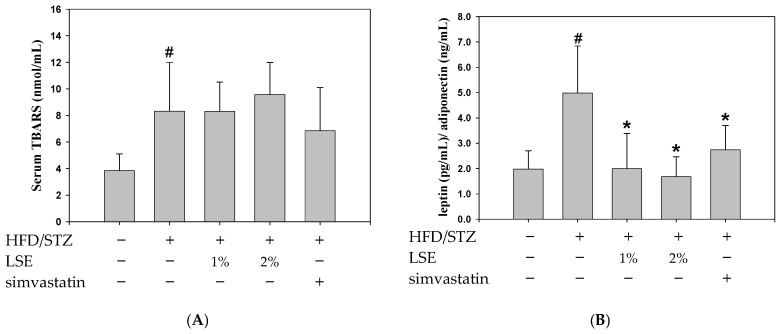

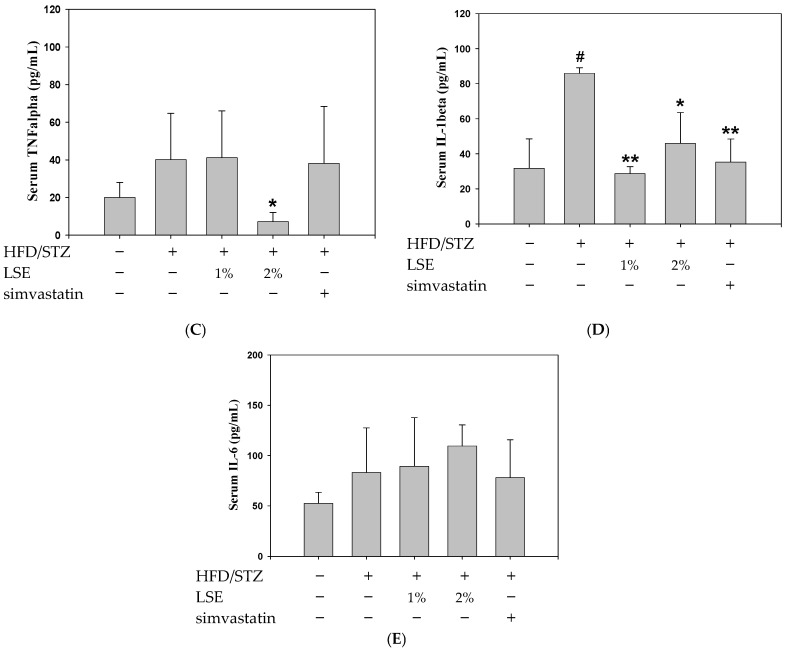
The effects of LSE on proinflammatory cytokines in mice following HFD/STZ induction. The MetS mice induced by HFD/STZ treatment (HFD for 12 weeks, followed by intraperitoneal injection of 40 mg/kg STZ for 5 consecutive days) were treated for 6 weeks with either LSE (1% or 2% w/w in feed), simvastatin (30 mg/kg, oral gavage, once daily), or vehicle (control group). (**A**) The TBARS assay was used to measure the lipid peroxidation level. The amount of MDA (nmole/mg protein), a biomarker of lipid peroxidation, was applied to display TBARS activity in nanomoles per milligram of protein. (**B**–**E**) The leptin and adiponectin ratio (**B**) and the levels of proinflammatory cytokines, such as TNF-α (**C**), IL-1β (**D**), and IL-6 (**E**), were measured by ELISA. The quantitative data were shown as mean ± SD (n ≥ 3), derived from at least three independent biological replicates. ^#^ *p* < 0.05 compared with the control group; * *p* < 0.05, ** *p* < 0.01 compared with the group of HFD/STZ. MetS: metabolic syndrome; HFD/STZ: high-fat diet/streptozotocin; LSE: lotus seedpod extract; TBARS: thiobarbituric acid reactive substances; MDA: malondialdehyde; TNF-α: tumor necrosis factor-alpha; IL: interleukin; ELISA: enzyme-linked immunosorbent assay.

Next, the levels of several cytokines in the serum were examined, including lipid peroxidation biomarker (TBARS), adipokines (leptin and adiponectin), and the inflammatory cytokines (TNF-α, IL-1β, and IL-6) (Figure 2). In Figure 2A,B,D, the levels of serum TBARS, leptin/adiponectin ratio, and IL-1β were significantly higher in the HFD/STZ group compared to the control group. Treatment with LSE or simvastatin was associated with lower leptin/adiponectin ratio and reduced expression of proinflammatory cytokines, including TNF-α and IL-1β (Figure 2B–D); however, TBARS and IL-6 levels did not show apparent tendency (Figure 2A,E).

### 3.3. The Effects of LSE on Hepatic Histology, Hepatic Fibrosis, Lipid Peroxidation, and Antioxidant Enzymes in Mice Following HFD/STZ Induction

**Figure 3:**
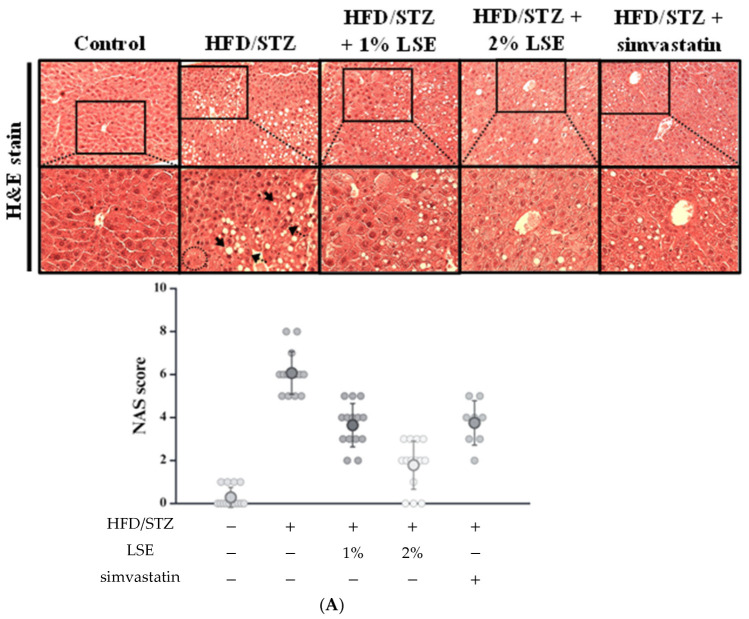

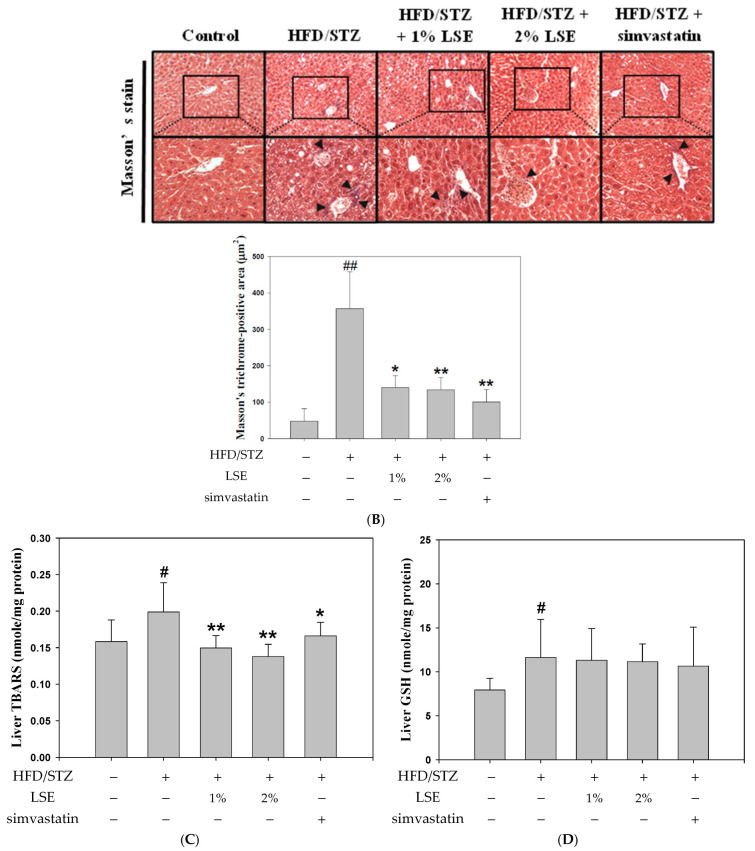

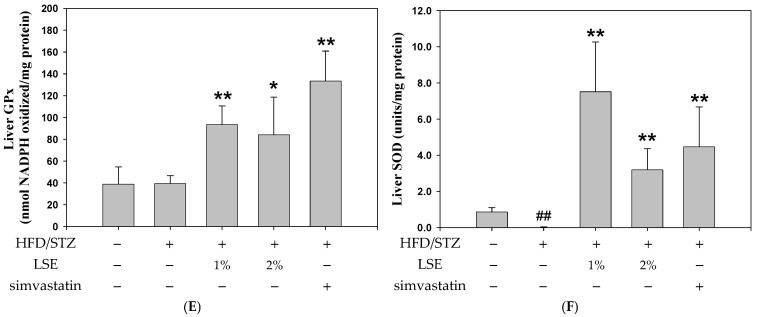
The effects of LSE on hepatic histology, hepatic fibrosis, lipid peroxidation, and antioxidant enzymes in mice following HFD/STZ induction. The MetS mice induced by HFD/STZ treatment (HFD for 12 weeks, followed by intraperitoneal injection of 40 mg/kg STZ for 5 consecutive days) were treated for 6 weeks with either LSE (1% or 2% *w*/*w* in feed), simvastatin (30 mg/kg, oral gavage, once daily), or vehicle (control group). (**A**) Representative images of H&E-stained liver sections from each group (100×, upper panel; 200×, lower panel) were used to evaluate hepatic tissue architecture. Macrovesicular and microvesicular steatosis (bold arrows) indicate the presence of multiple lipid droplets within hepatocytes. Clusters of inflammatory cell infiltration are marked by dotted line arrows. Hepatocyte hypertrophy (within circles) denotes enlarged hepatocytes that retain cytoplasmic features similar to those of surrounding steatotic cells. Hepatic lesions were assessed by NAS (lower panel), which includes steatosis (0–3), lobular inflammation (0–3), and hepato-cellular ballooning (0–2), with a total score ranging from 0 to 8. Each feature was assessed in randomly selected fields (n = 8–14) at 200× magnification by three independent observers blinded to the experimental groups. (**B**) Liver slices were stained with Masson’s trichrome (100×, upper panel; 200×, lower panel) to display the fibrotic tissue (indicated by arrows). The Masson’s stain-positive area in randomly selected fields were analyzed by image J (lower panel). The quantitative data of histological analysis were shown as mean ± SD (n = 8–14), derived from at least three independent biological replicates. (**C**) The hepatic level of lipid peroxidation was determined by MDA concentration (nmole/mg protein) in liver tissues using TBARS. (**D**–**F**) Hepatic GSH (D), GPx (E), and SOD (**F**) activities were analyzed by ELISA. The quantitative data were shown as mean ± SD (n ≥ 3), derived from at least three independent biological replicates. ^#^ *p* < 0.05, ^##^ *p* < 0.01 compared with the control group. * *p* < 0.05, ** *p* < 0.01 compared with the group of HFD/STZ. MetS: metabolic syndrome; HFD/STZ: high-fat diet/streptozotocin; LSE: lotus seedpod extract; NAS: NAFLD activity score; TBARS: thiobarbituric acid-reactive substances; MDA: malondialdehyde; GSH: glutathione; GPx: glutathione peroxidase; SOD: superoxide dismutase; ELISA: enzyme-linked immunosorbent assay.

To conduct a more thorough examination of the impact of LSE on liver histopathology and fibrosis, the liver tissues were analyzed using H&E and Masson’s stain. As shown in Figure 3A, abundant lipid droplets (bold arrows) were observed in hepatic tissues of the HFD/STZ group, which were markedly reduced following LSE treatment. Additionally, clusters of inflammatory cell infiltration (dotted arrows) and hepatocyte hypertrophy (within circles) were noted in the HFD/STZ group. To further evaluate liver pathology, the NAFLD activity score (NAS) was employed to assess hepatic steatosis, lobular inflammation, and hepatocellular ballooning. A total score of ≥5 is generally considered indicative of steatohepatitis (MASH), while a score of 0–2 suggests the early stage of MASLD. Moreover, an improvement of ≥2 points in NAS, without any worsening of fibrosis, is considered a valid indicator of histological improvement in the disease [31].

The NAS in the HFD/STZ group was 6.07 ± 0.99, indicating the presence of MASH. In contrast, NAS values were significantly lower in the LSE-treated groups (1%: 3.64 ± 1.01; 2%: 1.79 ± 1.21) and the simvastatin group (3.75 ± 1.04), reflecting reductions in steatosis, inflammatory cell infiltration, and hepatocellular ballooning (Figure 3A, lower panel). Figure 3B illustrates the expression of collagen, a key marker of fibrosis, as indicated by black arrows. The Masson’s-positive area, shown in blue, were quantified using Image J software (version 1.51k, NIH, Bethesda, MD, USA). Both LSE and simvastatin treatments led to a decrease in collagen accumulation induced by HFD/STZ. Altogether, these findings suggest that LSE effectively ameliorates HFD/STZ-induced hepatic steatosis and fibrosis.

The levels of TBARS and antioxidant enzymes in the hepatic tissues were measured to further evaluate the protective potential of LSE against HFD-/STZ-triggered metabolic syndrome (Figure 3C–F). LSE and simvastatin significantly suppressed the TBARS levels (Figure 3C), and also enhanced the activity of GPx and SOD in comparison to the group receiving HFD/STZ intervention (Figure 3E,F). Nevertheless, the HFD/STZ treatment led to elevated levels of glutathione (GSH), and there was no significance compared to the LSE or simvastatin groups (Figure 3D). These antioxidant properties may contribute to its anti-inflammatory effects, as oxidative stress is known to exacerbate inflammation and insulin resistance in MASLD [32].

### 3.4. The Effects of LSE on Dyslipidemia-, Hyperglycemia-, and Inflammation-Related Proteins in Mice Following HFD/STZ Induction

**Figure 4:**
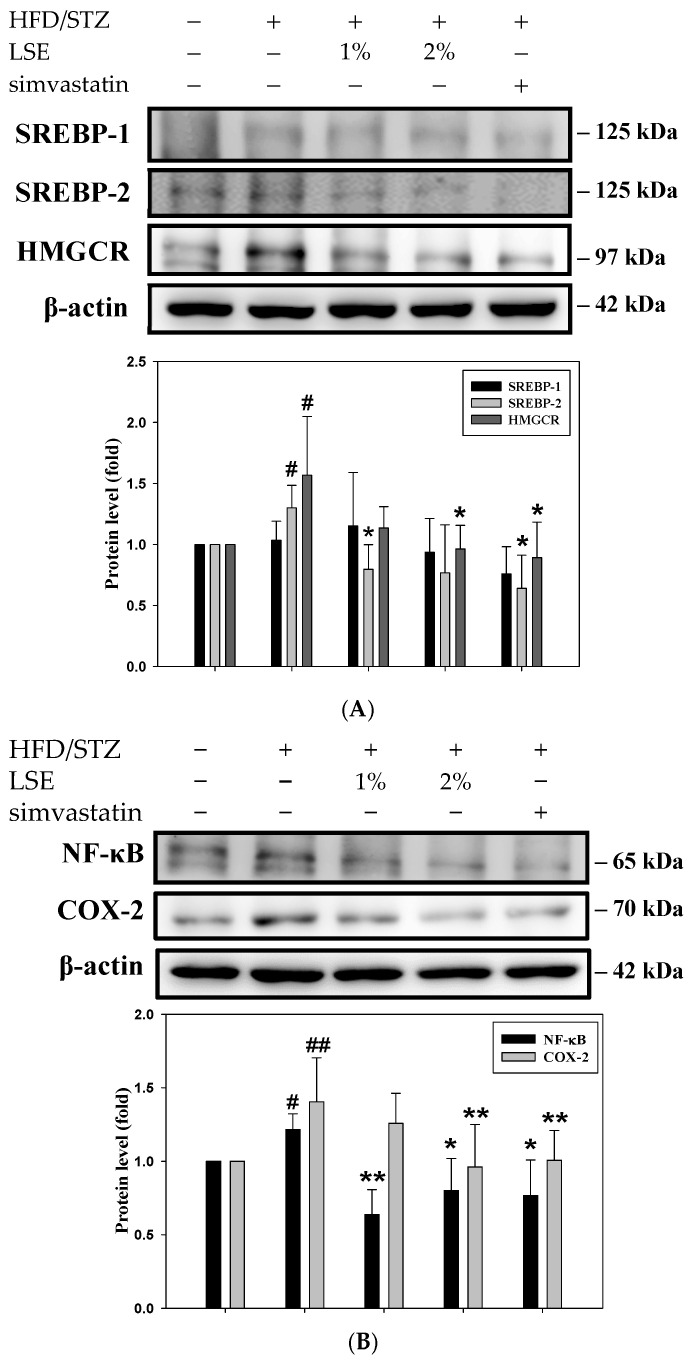

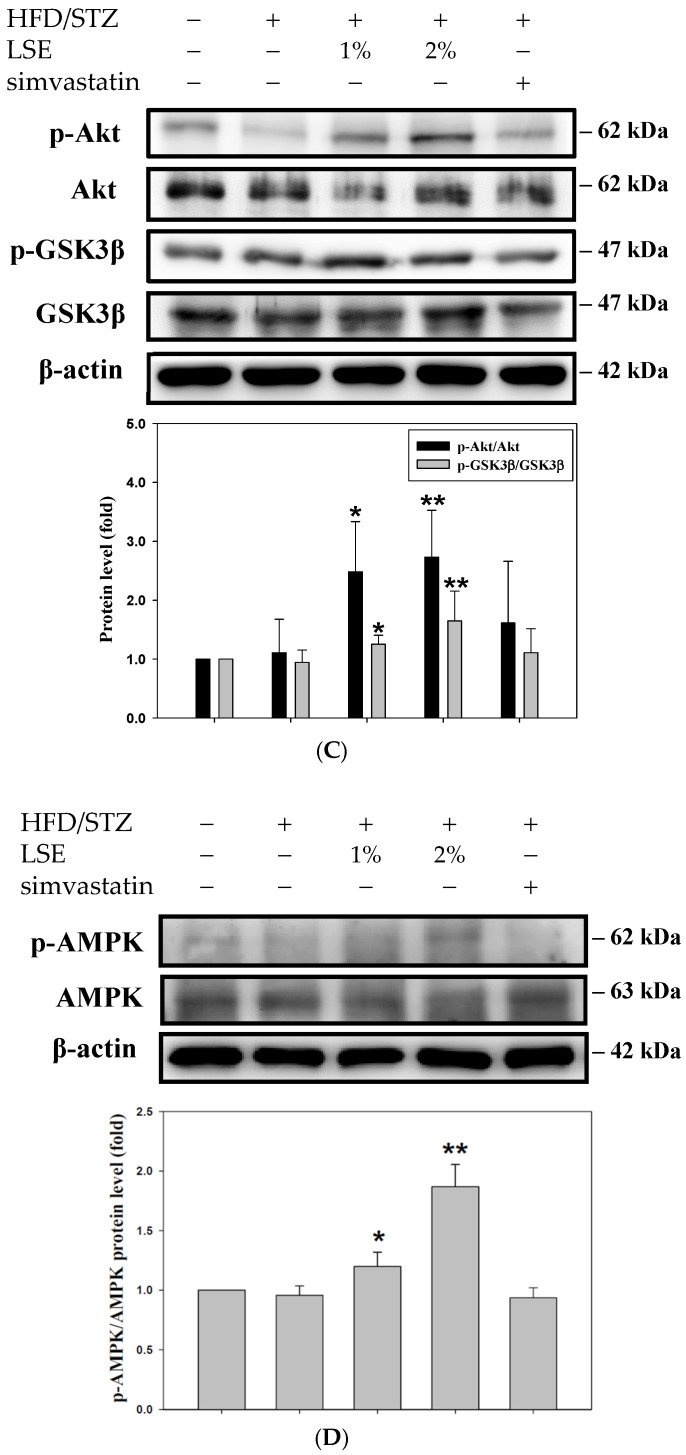
The effects of LSE on dyslipidemia-, hyperglycemia- and inflammation-related proteins in mice following HFD/STZ induction. The MetS mice induced by HFD/STZ treatment (HFD for 12 weeks, followed by intraperitoneal injection of 40 mg/kg STZ for 5 consecutive days) were treated for 6 weeks with either LSE (1% or 2% *w*/*w* in feed), simvastatin (30 mg/kg, oral gavage, once daily), or the vehicle (control group). The protein expressions of SREBP-1, SREBP-2, HMGCR (**A**), NF-κB, COX-2 (**B**), p-Akt, Akt, p-GSK3β, GSK3β (**C**), and p-AMPK and AMPK (**D**) were measured by Western blotting. β-actin performed as an internal control. The quantitative data of Western blot analysis were shown as mean ± SD (n ≥ 3), derived from at least three independent biological replicates. ^#^ *p* < 0.05, ^##^ *p* < 0.01 compared with the control group; * *p* < 0.05, ** *p* < 0.01 compared with the group of HFD/STZ. MetS: metabolic syndrome; HFD/STZ: high-fat diet/streptozotocin; LSE: lotus seedpod extract; SREBP: sterol regulatory element-binding protein; HMGCR: 3-hydroxy-3-methylglutaryl coenzyme A (HMG-CoA) reductase; NF-κB: transcription factor–nuclear factor kappa B; COX-2: cyclooxygenase-2; GSK3β: glycogen synthase kinase 3β; AMPK: adenosine monophosphate-activated protein kinase.

Subsequently, the signaling pathways of CHOL formation, glycogen synthesis, and inflammation were evaluated to examine the anti-MASLD effect of LSE. Figure 4A,B illustrated that HFD/STZ markedly raised the levels of SREBP-2, HMGCR, NF-κB, and COX-2, in contrast to LSE and simvastatin treatments lowered these values mentioned above. Compared to the HFD/STZ intervention, LSE also increased the expressions of p-Akt/Akt and p-GSK3β/GSK3β proteins (Figure 4C). These findings revealed that LSE mitigated dyslipidemia, hyperglycemia, and inflammation are caused by HFD/STZ. In addition, adenosine monophosphate-activated protein kinase (AMPK) has been reported to not only block CHOL and TG biosynthesis, but also modulate inflammation and oxidative stress in the liver [33]. The expression of AMPK was evaluated to better investigate the mediated mechanisms of upstream signaling. As illustrated in Figure 4D, LSE treatments resulted in higher levels of AMPK relative to HFD/STZ stimulation. AMPK activation is known to regulate mitophagy and improve mitochondrial integrity, which could prevent the redistribution of fatty acids to peripheral organs, including the liver [34].

### 3.5. The Effects of LSE and OA, Individually or Synergistically, on the Viability of HepG2 Cells

To clarify molecular mechanisms underlying the protective effect of LSE, an in vitro experiment was further performed. First, HepG2 cells were subjected to various dosages of OA (0, 0.1, 0.2, 0.5, 0.6, 0.8, and 1 mM) or LSE (0, 0.1, 0.5, 1, 5, 10, 50, and 100 µg/mL) over a period of 24 h to investigate the cytotoxicity. Figure S2A demonstrated a decrease in cell survival when HepG2 cells were incubated with OA at 0.8 and 1 mM; thus, 0.6 mM of OA was selected for the subsequent experiments due to its ability to maintain cell growth. In Figure S2B, there was a declining tendency when exposed to concentrations higher than 10 µg/mL. The HepG2 cells were challenged with 0.6 mM OA for 24 h, with or without non-cytotoxic dosages of LSE (1 and 5 μg/mL), in order to develop the model of lipid accumulation. Figure S2C showed no significance for cell viability within each group.

### 3.6. The Effects of LSE on Intracellular Lipid Deposition and Inflammatory Regulators in the OA-Induced HepG2 Cells

**Figure 5:**
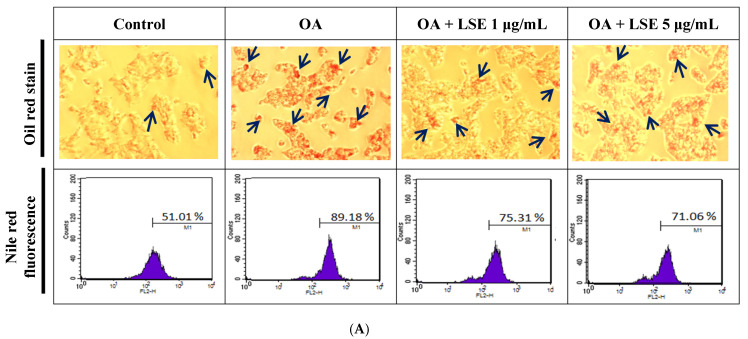

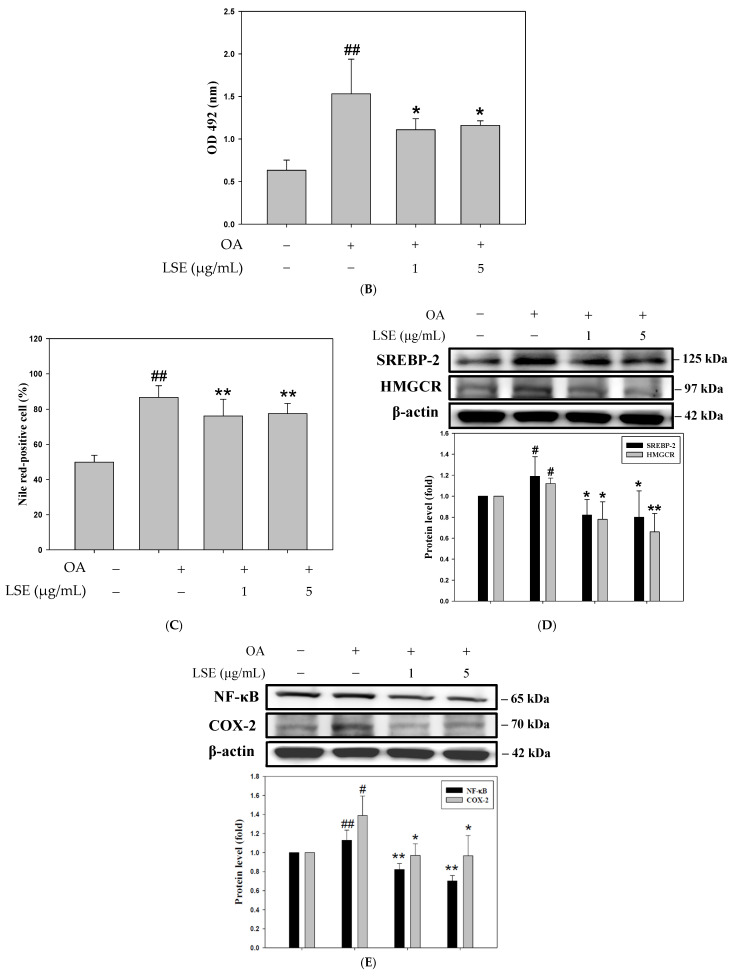
The effects of LSE on intracellular lipid deposition and inflammatory regulators in the OA-induced HepG2 cells. HepG2 cells were exposed to 0.6 mM OA in the presence or absence of specified doses of LSE (1 and 5 µg/mL) for 24 h. (**A**) Oil red was applied to the cells after treatments, and the stained cells were observed under a 100× microscope (upper panel). The stained lipid was identified by the red droplets clustered within the cells. Nile red staining was utilized to quantify the amount of intracellular fat via flow cytometric analysis (lower panel). (**B**) The oil red-stained culture dish was extracted with dye by adding 1 mL of isopropanol and then diluted 5× in ddH2O. The absorbance of the dye was recorded at 492 nm. (**C**) The Nile red values by flow cytometry were defined as the proportion of Nile red-positive cells in relation to all cell counts. The protein expressions of SREBP-2 and HMGCR (**D**), and NF-κB and COX-2 (**E**) were examined by Western blotting. β-actin performed as an internal control. The quantitative data were shown as mean ± SD (n ≥ 3), derived from at least three independent biological replicates. ^#^ *p* < 0.05, ^##^ *p* < 0.01 compared with control group; * *p* < 0.05, ** *p* < 0.01 compared with OA-induced group. OA: oleic acid; LSE: lotus seedpod extract; SREBP: sterol regulatory element-binding protein; HMGCR: 3-hydroxy-3-methylglutaryl coenzyme A (HMG-CoA) reductase; NF-κB: transcription factor–nuclear factor kappa B; COX-2: cyclooxygenase-2.

Oil red and Nile red, lipophilic dyes, were used to stain HepG2 cells in order to assess the effect of LSE on intracellular lipid deposits caused by OA. As shown in Figure 5A (upper panel), the lipid droplets in red were considerably observed in OA-stimulated HepG2 cells, which were diminished by LSE at a low dose (1 μg/mL). Additionally, the culture medium dyed with oil red was further extracted and measured at 492 nm. In Figure 5B, the quantitative data revealed that the down-regulatory effect of LSE was comparable to the presence of oil red-stained cells detected by the microscope. In Figure 5A (lower panel), the intracellular lipid content was detected by Nile red labeling using flow cytometry. As illustrated in Figure 5C, the quantitative data demonstrated that OA caused the high intensity of fluorescence, which was suppressed by LSE. The results showed that non-cytotoxic dosages of LSE decreased OA-induced deposits of lipids in HepG2 cells.

To further explore the LSE-mediated lipid and inflammatory regulators in OA-treated HepG2 cells, related protein expressions were assessed by Western blotting. Figure 5D,E illustrated that the levels of SREBP-2, HMGCR, NF-κB, and COX-2 were increased by OA stimulation, which were downregulated by LSE. These results indicate that LSE inhibited OA-upregulated CHOL formation and inflammation.

### 3.7. The Effects of LSE on ROS Production, Insulin Resistance, and Insulin Signaling in OA-Induced HepG2 Cells

**Figure 6:**
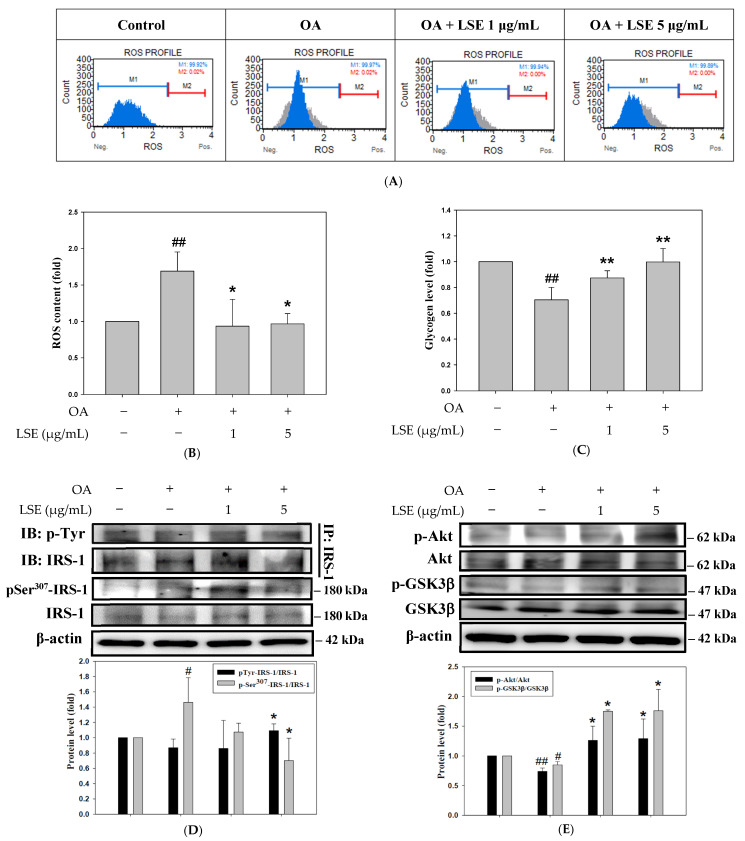

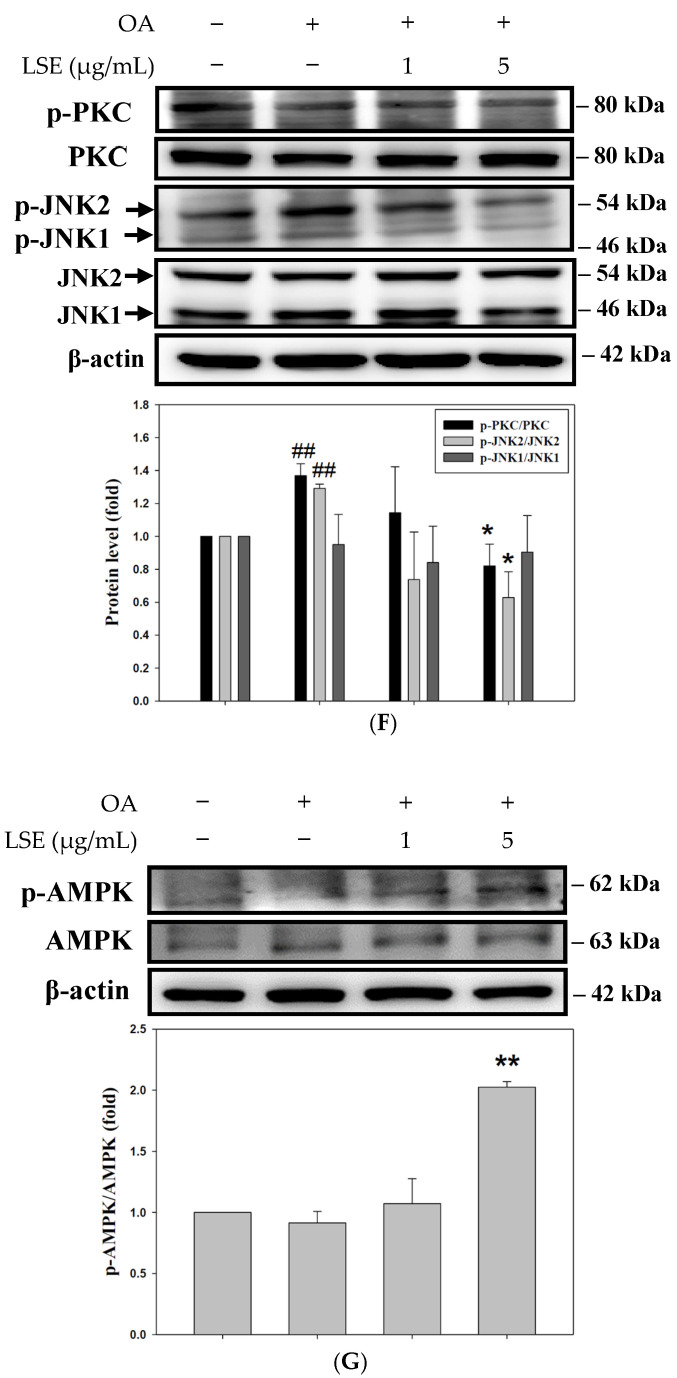
The effects of LSE on ROS production, insulin resistance, and insulin signaling in the OA-induced HepG2 cells. HepG2 cells were exposed to 0.6 mM OA in the presence or absence of specified doses of LSE (1 and 5 µg/mL) for 24 h. (**A**) DCFH-DA staining was utilized to evaluate the ROS using flow cytometry. (**B**) ROS content were expressed as percentage of DCF-positive cells relative to the total cell count. (**C**) The cellular glycogen content was assayed by ELISA. (**D**) Cell extracts were immunoprecipitated (IP) with an IRS-1 antibody. The precipitated complexes were examined for immunoblotting (IB) using p-Tyr or IRS-1 antibodies. The protein levels of pSer307-IRS-1 and IRS-1 were also determined by Western blotting. The protein expressions of p-Akt, Akt, p-GSK3β, GSK3β (**E**), p-PKC, PKC, p-JNK, JNK (**F**), and p-AMPK and AMPK (**G**) were determined by Western blotting. β-actin performed as an internal control. The quantitative data were shown as mean ± SD (n ≥ 3), derived from at least three independent biological replicates. ^#^ *p* < 0.05, ^##^ *p* < 0.01 compared with control group; * *p* < 0.05, ** *p* < 0.01 compared with OA-induced group. OA: oleic acid; LSE: lotus seedpod extract; DCFH-DA: 2′,7′-dichlorofluorescin diacetate; ROS: reactive oxygen species; ELISA: enzyme-linked immunosorbent assay; GSK3β: glycogen synthase kinase 3β; IRS-1: insulin receptor substrate-1; PKC: protein kinase C; JNK: c-Jun N-terminal kinases; AMPK: adenosine monophosphate-activated protein kinase.

In order to evaluate how LSE improved insulin resistance and oxidative stress caused by OA, the cellular ROS content and glycogen synthesis were further measured. The fluorescence intensity of HepG2 cells labeled with DCFH-DA was assessed by flow cytometry (Figure 6A). Figure 6B showed that LSE suppressed the OA-caused ROS production. Also, the level of glycogen was reduced by OA treatment, which was raised by LSE (Figure 6C). To further clarify the mechanism of the insulin-mediated signaling pathway, phosphorylation of IRS-1 was investigated using an immunoprecipitation (IP) assay. Previous studies have indicated that dysfunction of insulin signaling was attributed to the irregular phosphorylation of serine residues on IRS-1 [8]. As shown in Figure 6D, high-dose LSE treatment (5 μg/mL) significantly enhanced the complex of phosphorylated tyrosine and IRS-1 compared to the OA-treated group. In contrast, there was a decrease in the phosphorylation of serine residues on IRS-1 by LSE treatments. Collectively, the results revealed that LSE attenuated OA-stimulated oxidative stress and improved insulin resistance.

Subsequently, the factors of insulin signaling were examined using Western blotting. Figure 6E demonstrated that LSE elevated the expressions of p-Akt/Akt and p-GSKβ/GSKβ in comparison to OA treatment. It has been disclosed that the induction of PKC/JNK might induce insulin resistance and β-cell dysfunction [35]. LSE treatment (5 μg/mL) obviously inhibited the phosphorylation of PKC and JNK2, which were elevated by OA induction (Figure 6F). Also, the effect of LSE on AMPK, a key regulator of cellular energy metabolism, was assessed in OA-treated HepG2 cells. As shown in Figure 6G, high-dose LSE (5 μg/mL) apparently upregulated the activation of AMPK in comparison to the OA intervention.

### 3.8. The Effects of AMPK Inhibitor on the LSE-Corrected Glycogen Synthesis, Inflammation, and Lipid Accumulation in OA-Induced HepG2 Cells

**Figure 7:**
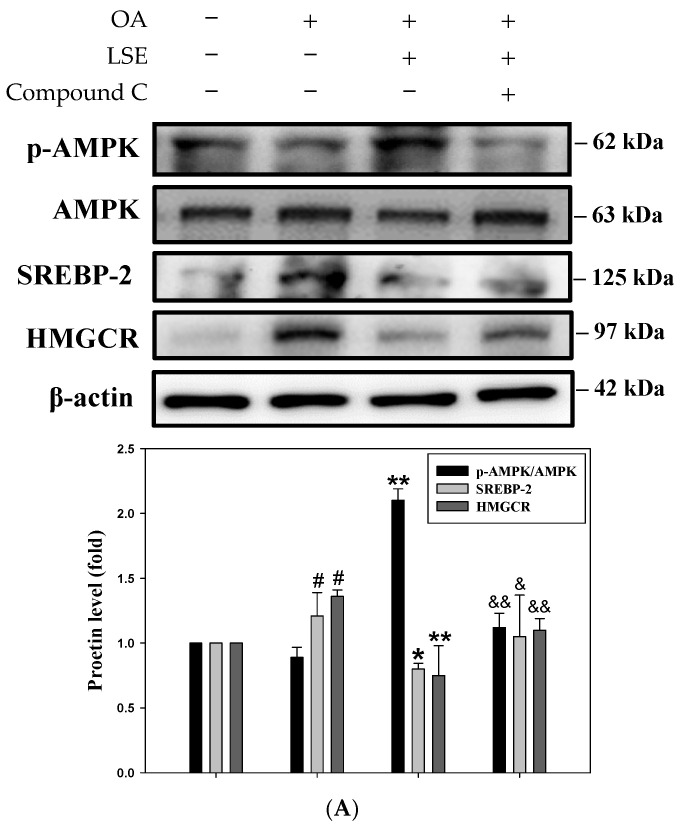

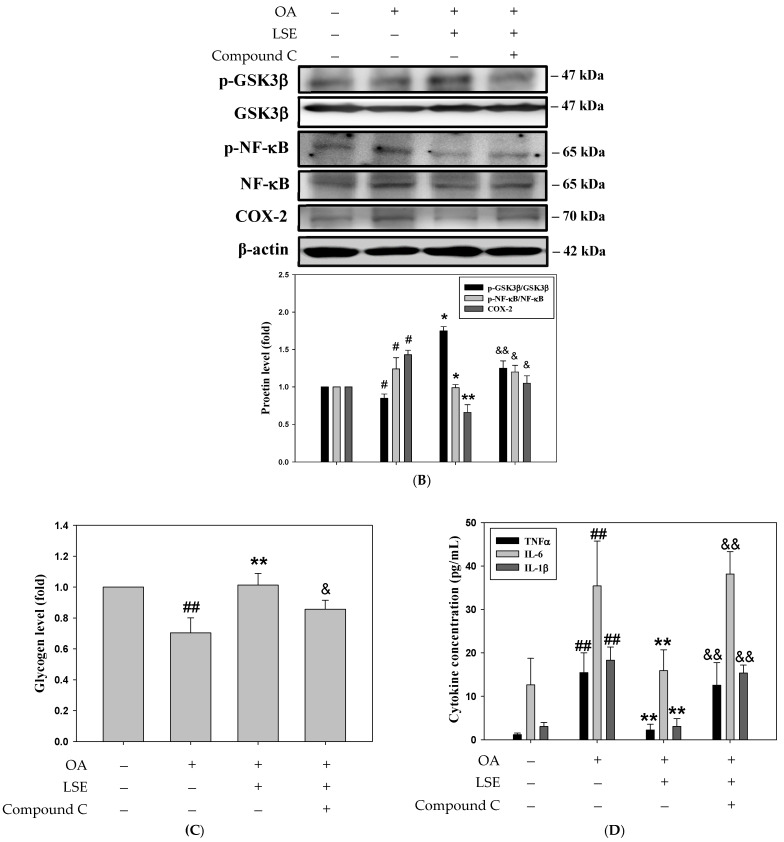

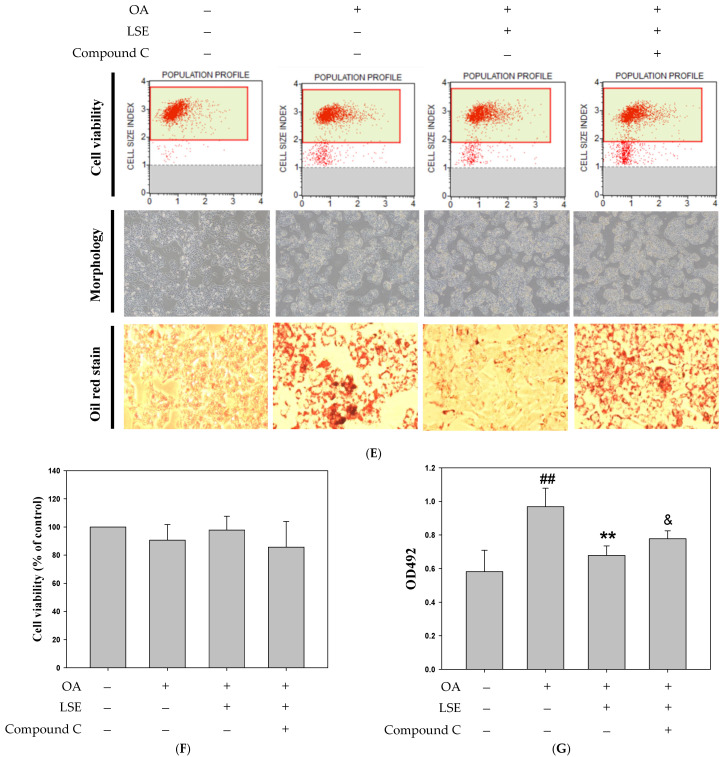
The effects of AMPK inhibitor on the LSE-corrected glycogen synthesis, inflammation, and lipid accumulation in the OA-induced HepG2 cells. HepG2 cells were underwent pre-treatment with or without compound C (3 μM), followed by treatment with LSE (5 μg/mL) in the presence of OA (0.6 mM) for 24 h. The protein expressions of p-AMPK, AMPK, SREBP-2, and HMGCR (**A**), p-GSK3β, GSK3β, p-NF-κB, NF-κB, and COX-2 (**B**) were determined by Western blotting. β-actin performed as an internal control. (**C**) The cellular glycogen content was assayed by ELISA. (**D**) The levels of proinflammatory cytokines, such as TNF-α, IL-6, and IL-1β, were detected by ELISA kits. (**E**) The cell viability was measured by PI staining using flow cytometry (upper panel), and the cell morphology was photographed under a light microscope (100×, middle panel). The cells were also stained with oil red and then observed under a 100× microscope (lower panel). The red droplet assembled in the cell were indicated as the stain lipid. (**F**) The quantitative data of cell viability were presented as the percentage of the control, which was set as 100%. (**G**) The oil red-stained culture dish was extracted with dye by adding 1 mL of isopropanol and then diluted 5× in ddH2O. The absorbance of the dye was recorded at 492 nm. The quantitative data were shown as mean ± SD (n ≥ 3), derived from at least three independent biological replicates. ^#^ *p* < 0.05, ^##^ *p* < 0.01 compared with control; * *p* < 0.05, ** *p* < 0.01 compared with OA-induced group. ^&^ *p* < 0.05, ^&&^ *p* < 0.01 compared with the group of OA plus LSE. OA: oleic acid; LSE: lotus seedpod extract; AMPK: adenosine monophosphate-activated protein kinase; SREBP: sterol regulatory element-binding protein; HMGCR: 3-hydroxy-3-methylglutaryl coenzyme A (HMG-CoA) reductase; GSK3β: glycogen synthase kinase 3β; NF-κB: transcription factor–nuclear factor kappa B; COX-2 cy-clooxygenase-2; ELISA: enzyme-linked immunosorbent assay; TNF-α: tumor necrosis factor-alpha; IL: interleukin, PI: propidium iodide.

In order to verify that the activation of AMPK is responsible for the protective effect of LSE on HepG2 cells treated with OA, the AMPK inhibitor compound C was utilized in the subsequent experiments. First, Western blotting was performed to evaluate the expression levels of proteins related to glycogen synthesis, inflammation, and insulin signaling. In Figure 7A,B, the regulatory effects of LSE on the above-mentioned proteins were attenuated by treatment with compound C, as evidenced by increased expression levels of SREBP-2, HMGCR, and COX-2, as well as the raised phosphorylation of NF-κB. Moreover, the LSE-induced increase in GSK3β phosphorylation was reversed by compound C. In the OA-stimulated cells was also confirmed that NF-κB phosphorylation was significantly elevated, indicating reduced glycogen synthase activity and enhanced activation of inflammatory signaling pathways. Also, compound C reversed the LSE-mediated effects on the production of glycogen and proinflammatory cytokines (Figure 7C,D). Furthermore, the cell viability analysis and oil red staining were analyzed using flow cytometry under pre-treatment with compound C (Figure 7E). There was no noticeable change in cell viability, even following compound C pre-treatment (Figure 7F). Nonetheless, the fluorescence intensity of oil red was reduced by pre-treatment with compound C, thereby diminishing the inhibitory effect of LSE. (Figure 7G). These findings indicated that LSE exhibited protective effects on insulin signaling and lipid metabolism by specifically targeting AMPK.

## 4. Discussion

Despite the increasing global burden of MASLD, targeted pharmacological therapies remain unavailable [36]. Although lifestyle interventions are considered fundamental, dietary habits play a critical role in the pathogenesis and potential prevention of MASLD [3]. High-fat and high-sugar diets contribute significantly to the onset of insulin resistance, adipose tissue dysfunction, and hepatic lipid accumulation, which are central to the “multiple-hit” hypothesis of MASLD. Excessive caloric intake, particularly of saturated fats and refined carbohydrates, promotes de novo lipogenesis and impairs lipid oxidation, thereby leading to hepatic steatosis and inflammation [37]. Conversely, dietary interventions, including increased consumption of polyphenol-rich foods, dietary fiber, and unsaturated fatty acids, have been shown to improve metabolic profiles and reduce hepatic fat content [38]. Natural compounds derived from herbs and plant extracts have also demonstrated potential benefits in the management of MetS [39]. Given the adverse effects and low adherence associated with current medications [40], the development of safe, well-tolerated supplements represents a promising therapeutic strategy.

In the present study, mice were fed an HFD combined with low-dose STZ injection exhibited hallmark features of MetS and MASLD. These included increased body weight (Figure 1B), insulin resistance (Figure 1D), elevated blood glucose (Figure 1E), and disordered lipid profiles (Figure S1A), indicating that the combination model effectively recapitulated the pathological features such as hyperglycemia, insulin resistance, and dyslipidemia. The progression from MASLD to MASH is driven by both metabolic stress and chronic inflammation. It typically begins with lipid overload and hepatocyte damage, followed by immune cell activation and cytokine release, such as TNF-α and IL-6, which contribute to sustained inflammation and fibrosis [41]. Mitochondrial dysfunction contributes to oxidative stress and further promotes inflammation in MASLD by activating NF-κB signaling and hepatic immune cells (stellate/Kupffer cells), leading to the upregulation of proinflammatory cytokines [42]. In our model, these pathogenic events were reflected by elevated proinflammatory cytokine levels (Figure 2), as well as histopathological features including hepatic steatosis, inflammatory infiltration, and fibrotic deposition, as shown by H&E and Masson’s trichrome staining (Figure 3A,B). These findings were consistent with previous reports showing that the HFD/STZ model effectively mimics both the metabolic abnormalities of T2DM and the hepatic pathology of MASLD/MASH [2,3,4,21]. Therefore, our results validate the relevance and translational potential of this model for investigating the pathophysiological mechanisms and therapeutic strategies targeting T2DM and MASLD.

These pharmacological effects of LSE are likely linked to its rich phytochemical composition, particularly its high flavonoid content. As shown in Table S1, LSE contains a substantial amount of total flavonoids (86.4 ± 3.6%) and polyphenols (45.3 ± 9.5%), with quercetin-3-glucuronide (Q3G) being the most abundant individual compound (122.44 ± 2.24 mg/100 g DW), followed by isorhamnetin-3-glucuronide and myricetin-3-galactoside. These compounds are well-documented in the literature for their anti-inflammatory, antioxidant, and metabolism-regulating properties. In particular, Q3G has been reported to ameliorate endothelial insulin resistance through inhibition of reactive oxygen species-associated inflammation [43], supporting the biological effects observed in this study. The phytochemical profile of LSE therefore indicates its multifunctional efficacy in modulating the hepatic disturbances characteristic of MetS or MASLD.

In this study, simvastatin, a statin drug commonly used in clinical practice, was employed as the reference treatment. Statins are widely used as lipid-lowering agents that primarily act by inhibiting HMGCR, leading to a reduction in LDL-c and an overall improvement in cardiovascular outcomes. However, emerging evidence has raised concerns about their potential effects on glucose metabolism, particularly in increasing insulin resistance and elevating blood glucose levels. Several studies have reported that statin use is associated with a modestly increased risk of new-onset T2DM [44]. Despite these adverse effects on glucose metabolism, the benefits of statins in reducing cardiovascular events often outweigh the risks. However, careful monitoring of blood glucose levels and glycemic control is recommended [45]. In this study, the HOMA-IR (Figure 1D) and blood glucose (Figure S1B) in the simvastatin group were significantly decreased compared with the HFD/STZ group, but there was no noticeable recovery in OGTT level (Figure 1E). Collectively, further research is warranted to clarify the precise mechanisms by which statins affect glucose metabolism and to explore potential therapeutic strategies. In addition, combining statins with agents that improve insulin sensitivity, such as metformin or AMPK activators, may provide a promising approach [46].

Importantly, these anti-inflammatory effects were accompanied by marked improvements in hepatic steatosis and lipid accumulation, as observed in biochemical analyses (Figure 2) and histological examination (Figure 3A), indicating that LSE may interrupt the inflammatory cascade linking lipid dysregulation to liver injury. Additionally, Masson’s trichrome staining confirmed that collagen deposition, a hallmark of fibrosis, was markedly decreased by LSE (Figure 3B). These findings were consistent with the reduced NASs in the LSE-treated groups, particularly in the 2% LSE group, supporting the conclusion that LSE prevents the progression from MASLD to MASH and reinforces its potential as a dietary intervention for early-stage MASLD. Mechanistically, the hepatoprotective effects of LSE appear to be mediated through modulation of several key molecular pathways involved in hepatic lipid and glucose metabolism. AMPK plays a central role, acting as a metabolic master switch that enhances fatty acid oxidation, inhibits lipogenesis, and improves insulin sensitivity [33]. AMPK activation has been shown to ameliorate MASLD primarily through increasing hepatic fatty acid oxidation and decreasing lipid synthesis [47]. One of the key downstream targets of AMPK is acetyl-CoA carboxylase (ACC). Phosphorylation of ACC by AMPK leads to its inactivation, resulting in reduced malonyl–CoA synthesis. The decrease relieves the inhibition of carnitine palmitoyl transferase 1 (CPT1), thereby reducing hepatic lipid content [48]. In addition, insulin resistance throughout the body is correlated with a decline in AMPK activity, which suggests that AMPK activation in adipose tissue may be critical to anti-MASLD [49]. In this study, LSE treatment significantly upregulated AMPK activity, which in turn downregulated lipogenic transcription factors such as SREBP-2 and HMGCR, thereby attenuating de novo lipid synthesis (Figure 4A,B and Figure 5D,E). Furthermore, LSE enhanced insulin signaling by reducing serine 307 phosphorylation of IRS-1, a modification known to impair insulin receptor downstream signaling [8]. This was accompanied by increased phosphorylation of Akt and downstream activation of GSK3β, leading to improved glycogen synthesis (Figure 6D,E). It is necessary to perform further studies to clarify the anti-MASLD properties of LSE on the glycogenesis pathway, fatty acid oxidation, and mitochondrial activity. These findings suggest that LSE may contribute to mitigating hepatic steatosis and oxidative stress, while also improving insulin sensitivity, potentially through mechanisms involving AMPK activation; however, further correlation analyses are needed to confirm the direct links between AMPK activation and these downstream effects.

Moreover, oxidative/nitrosative stress is widely acknowledged as a major contributor to hepatocellular injury in MASLD and plays a pivotal role in its progression to MASH. An imbalance between ROS/RNS production and antioxidant defenses leads to protein and lipid peroxidation, shifting cellular redox homeostasis toward a pro-oxidant state. In MASLD/MASH, elevated ROS/RNS levels result from mitochondrial dysfunction, free fatty acid oxidation, and proinflammatory cytokine signaling, ultimately exacerbating liver injury [32]. Notably, LSE treatment significantly reduced intracellular ROS levels (Figure 6A), which are typically elevated in MASLD and contribute to mitochondrial impairment and inflammation. The antioxidant effects of LSE may help preserve hepatocyte viability and mitigate cytokine-induced hepatic damage, as reflected by the decreased expression of proinflammatory mediators such as TNF-α and IL-6 (Figure 2C,D). Importantly, both in vivo and in vitro results consistently indicated that LSE activates AMPK signaling, supporting its central role in mediating the observed metabolic improvements. In the HFD/STZ-induced mouse model, LSE upregulated hepatic AMPK expression (Figure 4D), which was accompanied by reduced expression of lipogenesis-related proteins such as SREBPs and HMGCR (Figure 4A), improved insulin signaling as evidenced by increased p-Akt and p-GSK3β (Figure 4C), and decreased hepatic inflammatory markers including NF-κB and COX-2 (Figure 4B). Parallel findings were observed in OA-treated HepG2 cells, where LSE enhanced AMPK activation (Figure 6G), suppressed ROS generation (Figure 6B), increased glycogen synthesis (Figure 6C), and restored insulin receptor substrate signaling by reducing Ser307 phosphorylation of IRS-1 (Figure 6D). Furthermore, the reversal of LSE’s beneficial effects by the AMPK inhibitor compound C (Figure 7) further confirmed the essential role of AMPK in mediating these protective actions. This consistent pattern across both in vivo and in vitro models strongly reinforces the mechanistic link between AMPK activation and the therapeutic potential of LSE in MASLD.

Several previous studies have demonstrated the beneficial effects of lotus seedpod-derived polyphenols, including oligomeric procyanidins (LSOPC) and flavonoid-rich extracts, in both in vitro and in vivo models of metabolic dysfunction. As summarized in Table S3, LSOPC administration in HFD-induced or HFD/STZ-induced diabetic rodent models effectively attenuated hepatic inflammation, oxidative stress, and lipid dysmetabolism, primarily through mechanisms involving RAGE-MAPK-NF-κB, and inflammatory cytokines such as IL-6 and TNF-α [50,51]. These findings are consistent with our results, wherein LSE supplementation ameliorated hepatic steatosis and fibrosis in HFD/STZ-induced mice (Figure 3), alongside modulation of insulin signaling and a reduction in pro-inflammatory markers (Figure 6D and Figure 7D). Moreover, previous cell-based studies using HepG2 cells exposed to OA or lipopolysaccharide (LPS) have confirmed the capacity of LSE and its bioactive constituents, such as epigallocatechin (EGC), to reduce lipid accumulation, oxidative damage, and mitochondrial apoptosis through ROS-related signaling pathways [20,52]. Our study further builds on this evidence by incorporating both in vivo and in vitro systems, and by elucidating the involvement of AMPK signaling as a convergent mechanism mediating the hepatoprotective effects of LSE. Compared to earlier studies, our findings offer a more comprehensive view of LSE’s impact on hepatic lipid metabolism and fibrosis, which are critical features of MASLD progression. Collectively, these findings not only support prior evidence of the metabolic benefits of lotus-derived polyphenols but also highlight the therapeutic potential of LSE as a plant-derived nutraceutical for MASLD intervention.

The promising outcomes observed with LSE administration underscore its potential as a dietary supplement or adjunct therapy for MASLD. LSE, which is rich in flavonoids, exhibits a broad range of bioactivities, including antioxidant, anti-inflammatory, anti-diabetic, and hepatoprotective effects. Given the lack of approved pharmacological treatments for MASLD and the limitations of existing drugs such as statins, LSE may offer a safer, natural alternative or complement to current management strategies. Although this study primarily focuses on mechanistic insights in in vivo and in vitro models, the use of an aqueous extract without organic solvents supports its feasibility for dietary applications. These findings provide a strong foundation for future research aimed at evaluating LSE’s efficacy, safety, and formulation as a dietary supplement, particularly for early-stage MASLD or for supporting metabolic health in high-risk populations.

## 5. Conclusions

**Figure 8:**
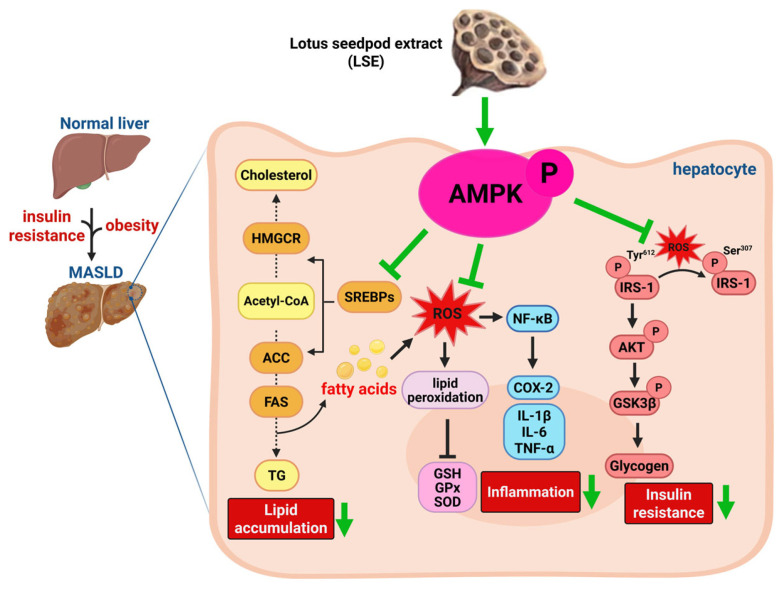
Overview of the inhibitory effects of LSE on hepatic carbohydrates and lipid dysmetabolism by targeting AMPK in vivo and in vitro. LSE functions the anti-MASLD effects via the inhibition of hepatic lipogenesis-regulatory factors, and the reduction in inflammatory factors/cytokines, as well as the induction of p-Tyr-IRS/Akt/GSK3β pathways that subsequently reduce the lipid accumulation, inflammation and insulin resistance by targeting AMPK. LSE: lotus seedpod extract; MASLD: metabolic dysfunction-associated steatotic liver disease; AMPK: adenosine monophosphate-activated protein kinase; HMGCR: 3-hydroxy-3-methylglutaryl coenzyme A (HMG-CoA) reductase; SREBPs: sterol regulatory element-binding proteins; ACC: acetyl-CoA carboxylase; FAS: fatty acid synthase; TG: triglycerides; ROS: reactive oxygen species; GSH: glutathione; GPx: glutathione peroxidase; SOD: superoxide dismutase; NF-κB: transcription factor–nuclear factor kappa B; COX-2 cy-clooxygenase-2; IL: interleukin; TNF-α: tumor necrosis factor-alpha; IRS-1: insulin receptor substrate-1; GSK3β: glycogen synthase kinase 3β.

In conclusion, this study demonstrates that flavonoid-rich lotus seedpod extract (LSE) exerts potent protective effects against high-fat diet and streptozotocin (HFD/STZ)-induced metabolic syndrome (MetS) and metabolic dysfunction-associated steatotic liver disease (MASLD) in mice. LSE ameliorated key features of MetS, including obesity, insulin resistance, dyslipidemia, hypertension, and systemic inflammation. In the liver, LSE significantly attenuated steatosis, fibrosis, and oxidative stress, as evidenced by improvements in histological architecture, NASs, collagen deposition, and antioxidant enzyme activities. Mechanistically, LSE activated AMPK signaling, suppressed the expression of lipogenesis- and inflammation-related proteins (SREBP-2, HMGCR; NF-κB, COX-2), and enhanced insulin signaling by restoring IRS-1/Akt/GSK3β pathways. In vitro experiments further confirmed that LSE reduced oleic acid (OA)-induced lipid accumulation, oxidative stress, and inflammatory responses in HepG2 cells, with these effects being largely dependent on AMPK activation. Collectively, these findings suggest that LSE is a promising natural compound with therapeutic potential for the management of MASLD/MASH, and highlight the importance of targeting AMPK-related metabolic and inflammatory pathways in liver disease treatment. Further studies are warranted to evaluate its clinical efficacy, bioactivity, and effects on mitochondrial function in adipose tissues (Figure 8).

## 6. Limitations

Although this study provides promising findings and mechanistic insights into the hepatoprotective effects of LSE, several limitations remain. First, while AMPK was identified as a central signaling node, the present study did not comprehensively explore its key downstream effectors such as phosphorylated ACC and CPT1, which are critical regulators of hepatic fatty acid oxidation and lipogenesis [48]. Additionally, potential interactions in modulating hepatic metabolism with other downstream pathways, including mTOR or PPARα/γ [53], were not investigated. These pathways may influence hepatic metabolism and warrant further exploration in future mechanistic studies. Second, the long-term safety, pharmacokinetic profile, and oral bioavailability of LSE were not evaluated. These parameters are crucial for the therapeutic development and clinical translation of botanical extracts and warrant further investigation in future preclinical and clinical studies. Third, the composition of other dietary ingredients was not adjusted to maintain isocaloric conditions across all groups, which may have slightly altered the overall nutrient balance and energy intake. Pair-feeding strategies or the use of isocaloric control diets in future studies would help disentangle the specific dietary contributions to metabolic outcomes. Lastly, although simvastatin was employed as a reference agent due to its established lipid-lowering and anti-inflammatory effects, no anti-diabetic drugs such as metformin or GLP-1 receptor agonists were included for comparison. The inclusion of such pharmacological agents would provide a more comprehensive benchmark for assessing LSE’s efficacy relative to standard therapeutic options for MASLD.

## Data Availability

All data of this study are included in this article.

## Supplementary Materials

The following supporting information can be downloaded at: https://www.mdpi.com/article/10.3390/antiox14050595/s1, Figure S1: Effects of LSE on the serum biochemical parameters of mice induced by a HFD/STZ treatment; Figure S2: Effects of LSE and OA, individually or synergistically, on the viability of HepG2 cells; Figure S3: The original Western blot of Figure 4A–D, Figure 5D,E, Figure 6D–G, and Figure 7A,B; Figure S4: The original H&E of Figure 3A and Masson’s stain of Figure 3B; Table S1: Composition of the lotus seedpod extracts (LSE); Table S2: Primary antibodies used for Western Blot analysis; Table S3: Comparison of the experimental model and dose of the referenced studies of lotus seedpod.

## Abbreviations

MetS	Metabolic syndrome
MASLD	Metabolic dysfunction-associated steatotic liver disease
HFD	High-fat diet
STZ	Streptozotocin
HOMA-IR	Homeostasis model assessment-insulin resistance index
OGTT	Oral glucose tolerance test
AMPK	Adenosine monophosphate-activated protein kinase
ROS	Reactive oxygen species
IRS-1	Insulin receptor substrate-1
PKB	Protein kinase B
GSK3β	Glycogen synthase kinase 3β
DM	Diabetes mellitus
NF-κB	Transcription factor–nuclear factor kappa B
TNF-α	Tumor necrosis factor-alpha
IL	Interleukin
FA	Fatty acids
TG	Triglycerides
CHOL	Cholesterol
HMGCR	3-hydroxy-3-methylglutaryl coenzyme A (HMG-CoA) reductase
SREBPs	Sterol regulatory element-binding proteins
LSE	Lotus seedpod extract
MDA	Malondialdehyde
TBARS	Thiobarbituric acid-reactive substances
GSH	Glutathione
GPx	Glutathione peroxidase
SOD	Superoxide dismutase
LDL	Low-density lipoprotein
HDL	High-density lipoprotein
GOT	Glutamic oxaloacetic transaminase
GPT	Glutamic pyruvic transaminase
BUN	Blood urea nitrogen
CRE	Creatinine
COX-2	Cyclooxygenase-2
OA	Oleic acid
PKC	Protein kinase C
JNK	c-Jun N-terminal kinases
ACC	Acetyl-CoA carboxylase
FAS	Fatty acid synthase
