# Supplementary material for: Protective Effects of Lotus Seedpod Extract on Hepatic Lipid and Glucose Metabolism via AMPK-Associated Mechanisms in a Mouse Model of Metabolic Syndrome and Oleic Acid-Induced HepG2 Cells
Source: Antioxidants (Basel). 2025 May 16;14(5):595. doi: 10.3390/antiox14050595 (PMC12108490; doi:10.3390/antiox14050595)
Supplement: Supplementary file 1 [file antioxidants-14-00595-s001.zip › antioxidants-3555568-supplementary.pdf]

Table S1. Composition of the lotus seedpod extracts (LSE)

Quantitative determination of composition		LSE (%)
Total polyphenol (Folin-Ciocalteu method)		45.3 ± 9.5
Total flavonoid (Jia method)		86.4 ± 3.6
Total anthocyanin (Fuleki and Francis method)		5.2 ± 1.7
Flavonoid compound of HPLC/ESI-MS-MS method[a]	Retention time (time)	Content (mg/100 g DW)[b]
Myricetin-3-galactoside	7.97	11.52 ± 2.16
Quercetin-3-glucuronide	8.72	122.44 ± 2.24
Isorhamnetin-3-glucuronide	9.58	30.27 ± 3.46
Isorhamnetin-3-glucoside	9.80	29.73 ± 4.94

[a] Phenolic compounds correspond to peaks as in by high-performance liquid chromatography-diode array detector (HPLC-DAD), liquid chromatography–mass spectrometry (LC-MS), and liquid chromatography-tandem mass spectrometry (LC-MS-MS) analysis chromatogram of 8 kinds of standard flavonoid compounds, including myricetin-3-galactoside, quercetin-3-glucuronide, isoquercitrin, isorhamnetin-3-glucuronide, isorhamnetin-3-glucoside, quercetin, kaempferol, and isorhamnetin. [b] Compounds were quantified froman external calibration of 7-methoxyflavanone (Sigma Chemical Co., St. Louis, MO,USA) in duplicate. The content was presented as mg/100 g dried weight (DW) of LSE.

Table S2. Primary antibodies used for Western Blot analysis

Antibody	Catalog No.	Source	Reactivity	Manufacturer	Phospho-site	Dilution
SREBP-1	sc-13551	Mouse	Mouse/Rat/Human	Santa Cruz Biotechnology	NA	1:500
SREBP-2	sc-13552	Mouse	Mouse/Rat/Human	Santa Cruz Biotechnology	NA	1:500
HMGCR	sc-271595	Mouse	Mouse/Rat/Human	Santa Cruz Biotechnology	NA	1:500
p-Akt	sc-7985	Rabbit	Mouse/Rat/Human	Santa Cruz Biotechnology	Ser474	1:500
Akt	sc-8312	Rabbit	Mouse/Rat/Human	Santa Cruz Biotechnology	NA	1:500
p-GSK3β	sc-11757	Rabbit	Mouse/Rat/Human	Santa Cruz Biotechnology	Ser9	1:500
GSK3β	sc-9166	Rabbit	Mouse/Rat/Human	Santa Cruz Biotechnology	NA	1:500
p-NF-κB	sc-136548	Mouse	Mouse/Rat/Human	Santa Cruz Biotechnology	Ser536	1:500
NF-κB	sc-8008	Mouse	Mouse/Rat/Human	Santa Cruz Biotechnology	NA	1:500
COX-2	sc-19999	Mouse	Mouse/Rat/Human	Santa Cruz Biotechnology	NA	1:500
AMPKα1/2	sc-74461	Mouse	Mouse/Rat/Human	Santa Cruz Biotechnology	NA	1:500
p-PKC	sc-377565	Mouse	Mouse/Rat/Human	Santa Cruz Biotechnology	Ser657	1:500
PKC	sc-17769	Mouse	Mouse/Rat/Human	Santa Cruz Biotechnology	NA	1:500
p-JNK	sc-6254	Mouse	Mouse/Rat/Human	Santa Cruz Biotechnology	Thr183/Tyr185	1:500
JNK	sc-7345	Mouse	Mouse/Rat/Human	Santa Cruz Biotechnology	NA	1:500
p-AMPKα	2535S	Rabbit	Mouse/Rat/Human	Cell Signaling Technology	Thr172	1:1000
β-actin	A5441	Mouse	Mouse/Rat/Human	Sigma-Aldrich	NA	1:10000

The table lists the primary antibodies used in this study, including their catalog numbers, source, species reactivity, manufacturing companies, and phosphorylation-specific sites where applicable, and working dilutions used for Western blot analysis.

Table S3. **Comparison of the experimental model and dose of the referenced studies of lotus seedpod**

Extract or polyphenol	Cell type or mouse strain	Cellular or animal model	Treatment condition[1] (dose, time)	Results	Mechanism of action	Reference
lotus seedpod oligomeric procyanidins (LSOPC)	Sprague-Dawley (SD) male rats	High-fat diet (HFD, 17.50% fat, mainly from lard, cholesterol, egg yolk powder, and 38% calories from fat)	0.2% or 0.5% (w/w) LSOPC for 12 weeks	Inhibition of AGE formation, liver inflammation, and oxidative stress.	(↑[2]) serum HDL-C<br>(↓[3]) TC, TG, FFA, AGEs, CML<br>(↓) RAGE/ MAPK<br>(↓) NF-κB, TNF-α, IL-1β, IL-6	J. Agric. Food Chem. 2015, 63, 6989–6998.
LSOPC	Male ICR mice	HFD (69% common diet, 10% lard, 15% sucrose, 5% egg yolk power, 1% cholesterol, and 0.2% sodium deoxycholate) plus STZ (45 mg/kg, continuously injected for 2 days)	LSOPC (150 mg/kg and 300 mg/kg) for 12 weeks	Improvement of glucose homeostasis, lipid metabolism, and heat generation in brown adipose tissue.<br>Inhibition of lipogenesis and gluconeogenesis in liver.	(↓) blood glucose, FFA, endotoxin<br>(↑) glucose uptake and glycolysis, thermogenesis<br>(↓) gluconeogenesis and lipogenesis	J. Agric. Food Chem. 2017, 65, 3801–3810
lotus seedpod extract (LSE) and epigallocatechin (EGC)<br>(cultivar: Dahe Lian)	HepG2 cells	Oleic acid (OA, 0.6 mM)	LSE (2.5, 5, and 10 μg/mL) or EGC (4 μM) for 48 h	Improvement of cell viability<br>Inhibition of intracellular lipid accumulation, oxidative stress and mitochondrial apoptosis pathway.	(↓) ROS, cytosol cyt. C, Bax<br>(↓) active-caspase3/8/9,	Nutrients 2019, 11, 2895.
LSE and EGC<br>(cultivar: Dahe Lian)	Male ICR mice<br>HepG2 cells	In vivo: lipopolysaccharide (LPS, 20 mg/kg)<br>In vitro: LPS (5 μg/mL)	In vivo: 1% or 2% (w/w) LSE<br>In vitro: LSE (1, 2.5 and 5 μg/mL) or EGC (2 μM) for 24 h.	Inhibition of inflammation.	(↓) TNF-α, IL-6, COX-2<br>(↓) NF-κB, iNOS, p38	Am J Chin Med. 2019, 47, 153–176.
LSE<br>(cultivar: Sheklian)	BALB/c mice<br>HepG2 cells	In vivo: HFD (61.9% fat as a percentage of total kilocalories) plus STZ (40 mg/kg, continuously injected for 5 days)<br>In vitro: OA (0.6 mM)	In vivo: 1% or 2% (w/w) LSE for 12 weeks<br>In vitro: LSE (1 and 5 μg/mL) for 24 h	Improvement of lipid and carbohydrate metabolism dysfunction.	(↓) body weight, epididymal fat<br>(↓) HOMA-IR, TNF-α, IL-1β<br>(↓) ROS, TBARS, IRS-1^{Ser307}<br>(↑) AMPK	In this study, 2025.

[1]Cells or mice responded to drug as follows under the treatment condition. [2]↑, induction of protein expression after treatment with extract. [3]↓, inhibition of protein expression after treatment with extract.

Figure S1

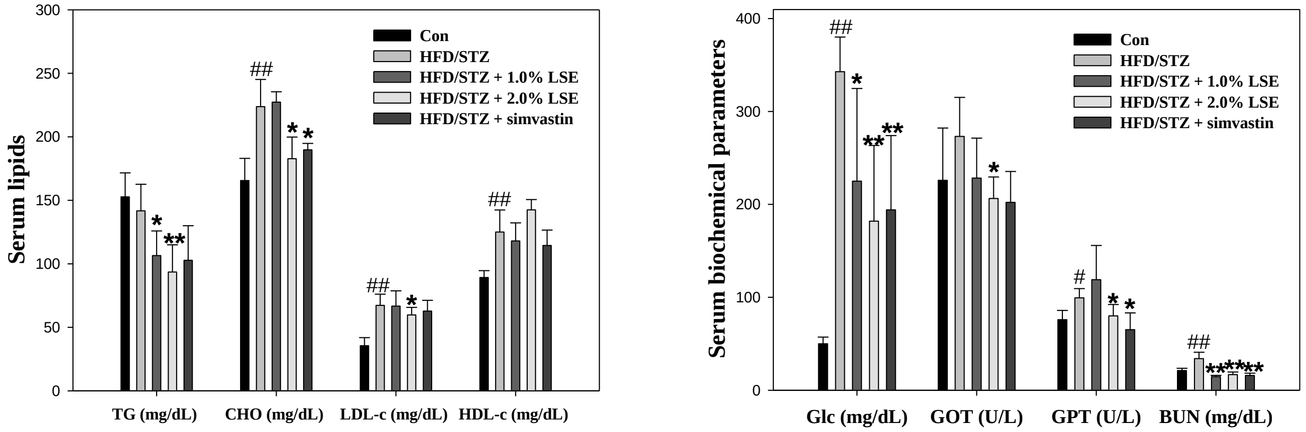

Figure S1. Effects of LSE on the serum biochemical parameters of mice induced by a HFD/STZ treatment. HFD combined with STZ treatment-induced the metabolism syndrome of mice were treated with LSE (1% and 2%) or statin for 6 weeks. The mice were sacrificed after 6 weeks, and serum was collected for analysis. The levels of serum lipids, including TG, CHO, LDL-c, and HDL-c **(A)**, as well as blood glucose (Glu) and liver/renal function markers (GOT, GPT, and BUN) **(B)**, were analyzed using a biochemical analyzer. The quantitative data were shown as mean ± SD (n ≥ 3), derived from at least three independent biological replicates. #p< 0.05, ##p< 0.01 compared with the control group. *p< 0.05, **p< 0.01 compared with the group of HFD plus STZ.

Figure S2

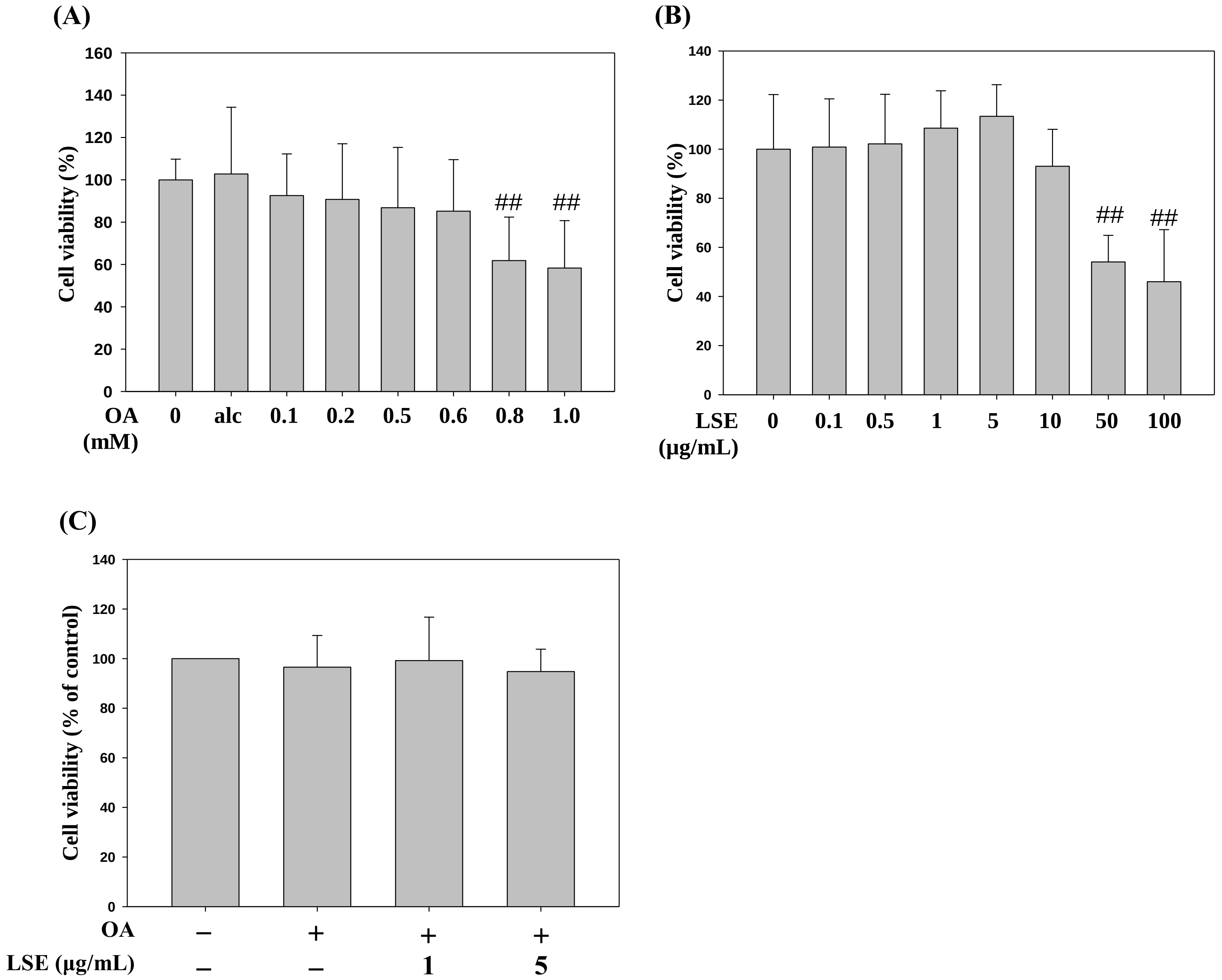

Figure S2. Effects of LSE and OA, individually or synergistically, on the viability of HepG2 cells. HepG2 cells were treated with various concentrations of OA (0.1-1.0 mM) **(a)** or LSE (0.1-100 μg/mL) **(b)** for 24 h. Alcohol (alc) served as a solvent control. **(c)** HepG2 cells were treated with 0.6 mM of OA in the presence or absence of indicated concentrations of LSE (1 and 5 μg/mL) for 24 h. Cell viability was analyzed by cell counting assay. The quantitative data were shown as mean ± SD (n ≥ 3), derived from at least three independent biological replicates. $^{\#}p < 0.01$ compared with the control.

Figure S3

Figure 4 (A)

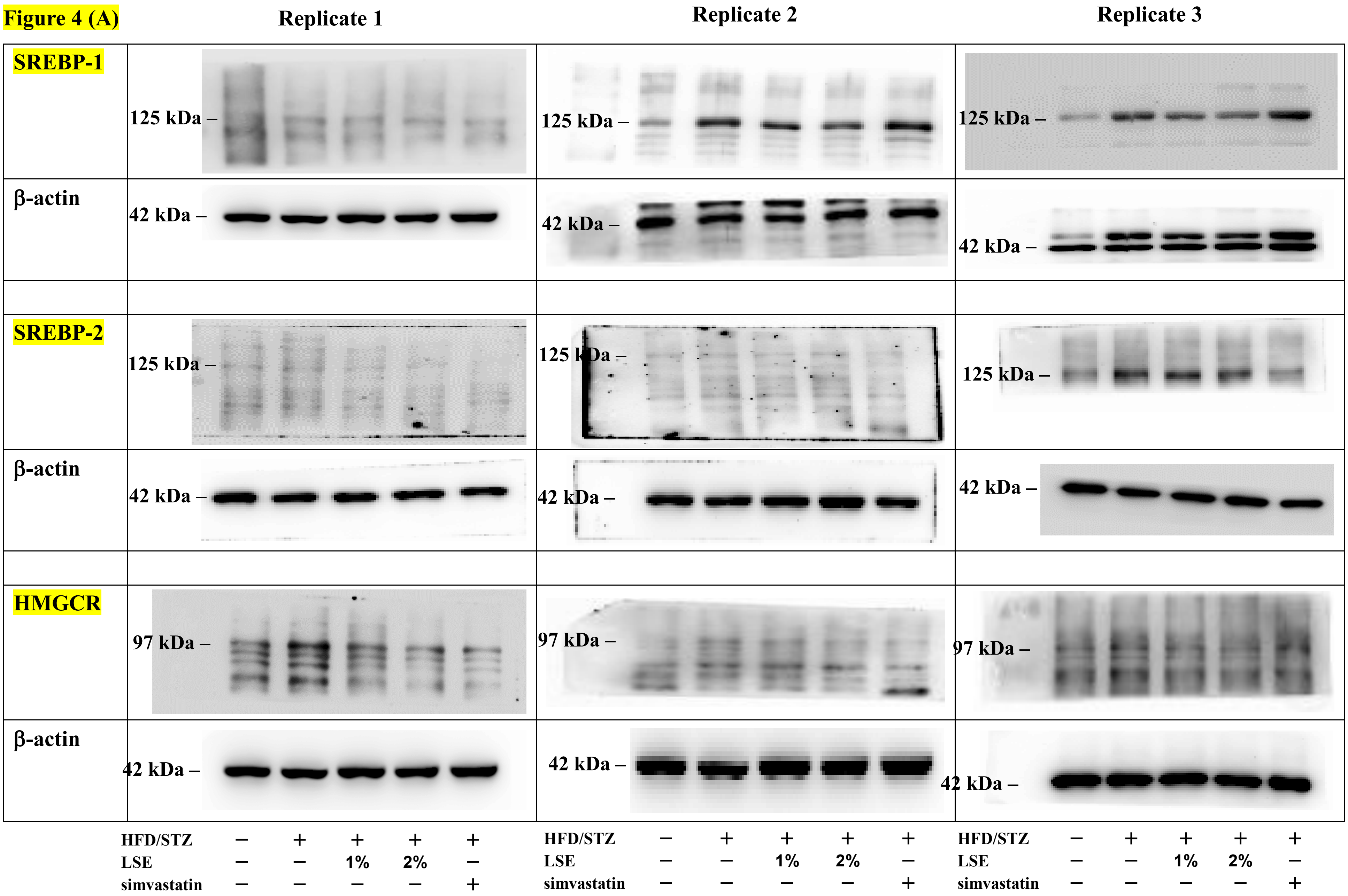

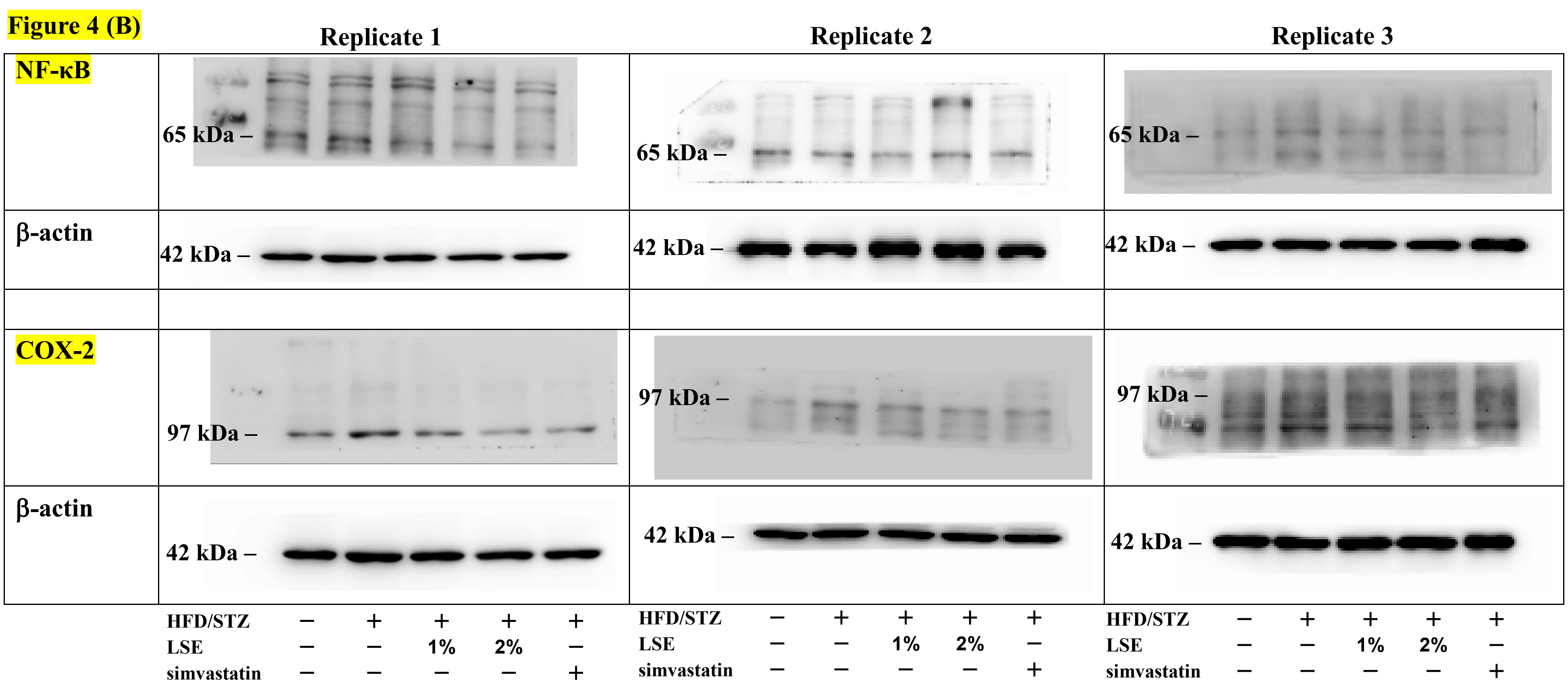
Figure 4 (B)
Replicate 1
Replicate 2
Replicate 3
NF-κB
65 kDa –
β-actin
42 kDa –
COX-2
97 kDa –
β-actin
42 kDa –
HFD/STZ – + + + +
LSE – – 1% 2% –
simvastatin – – – – +

Figure 4 (C)

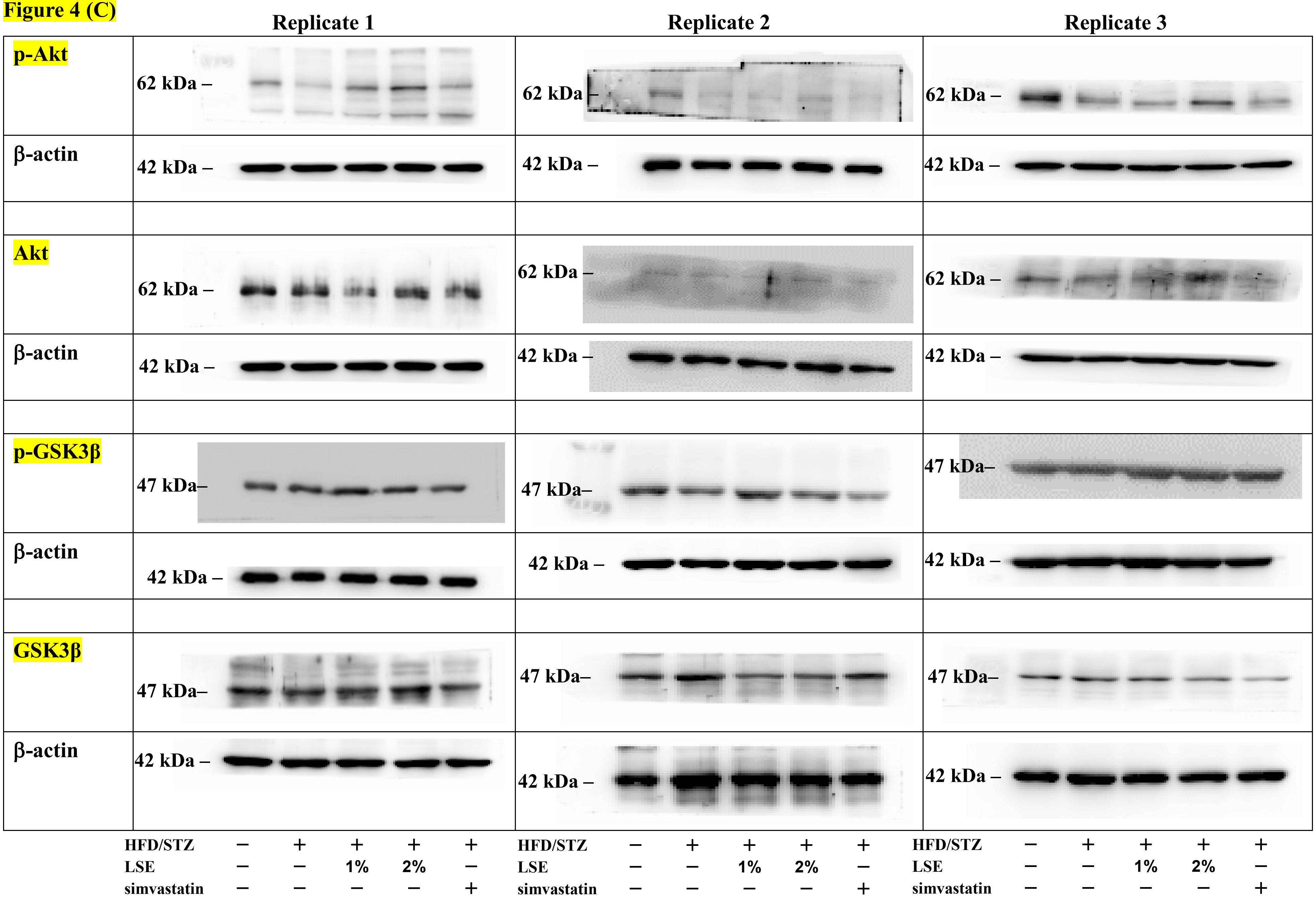

Replicate 1
Replicate 2
Replicate 3
p-Akt
62 kDa –
β-actin
42 kDa –
Akt
62 kDa –
β-actin
42 kDa –
p-GSK3β
47 kDa–
β-actin
42 kDa –
GSK3β
47 kDa–
β-actin
42 kDa –
HFD/STZ – + + + +
LSE – – 1% 2% –
simvastatin – – – – +

Figure 4 (D)

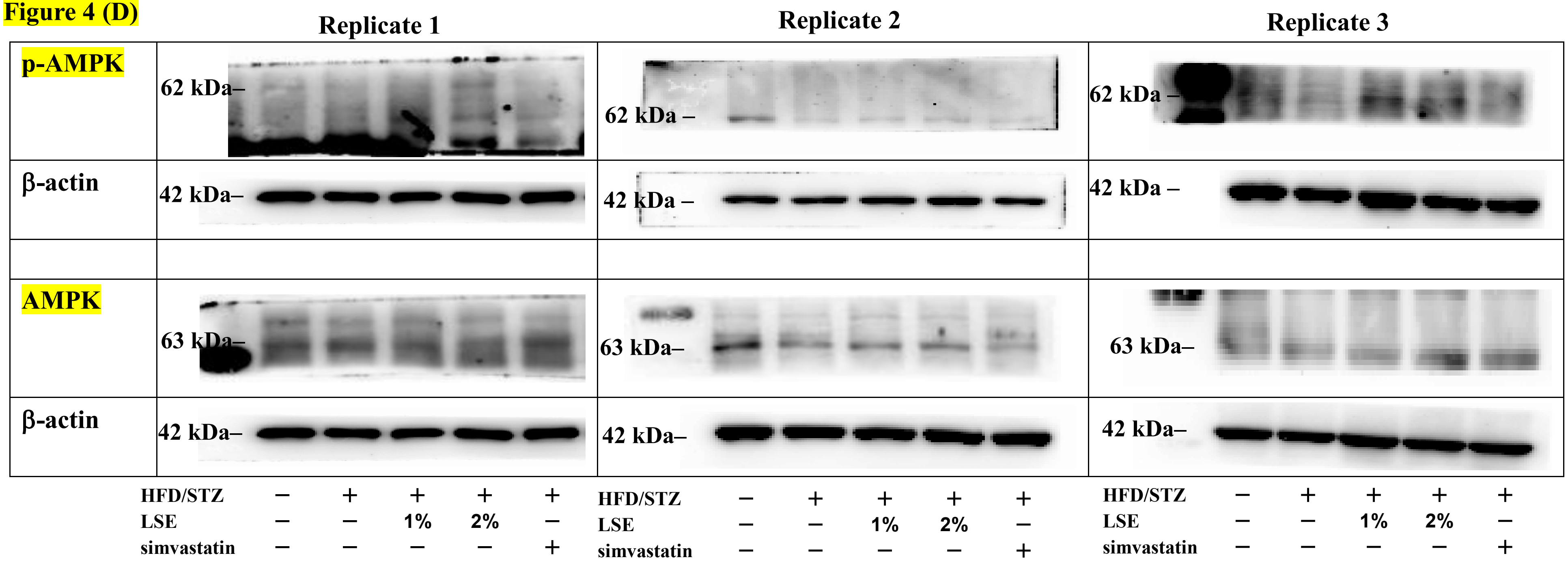
Replicate 1
Replicate 2
Replicate 3
p-AMPK
62 kDa–
62 kDa –
62 kDa –
β-actin
42 kDa–
42 kDa –
42 kDa –
AMPK
63 kDa–
63 kDa–
63 kDa–
β-actin
42 kDa–
42 kDa–
42 kDa–
HFD/STZ – + + + +
LSE – – 1% 2% –
simvastatin – – – – +
HFD/STZ – + + + +
LSE – – 1% 2% –
simvastatin – – – – +
HFD/STZ – + + + +
LSE – – 1% 2% –
simvastatin – – – – +

Figure 5 (D)

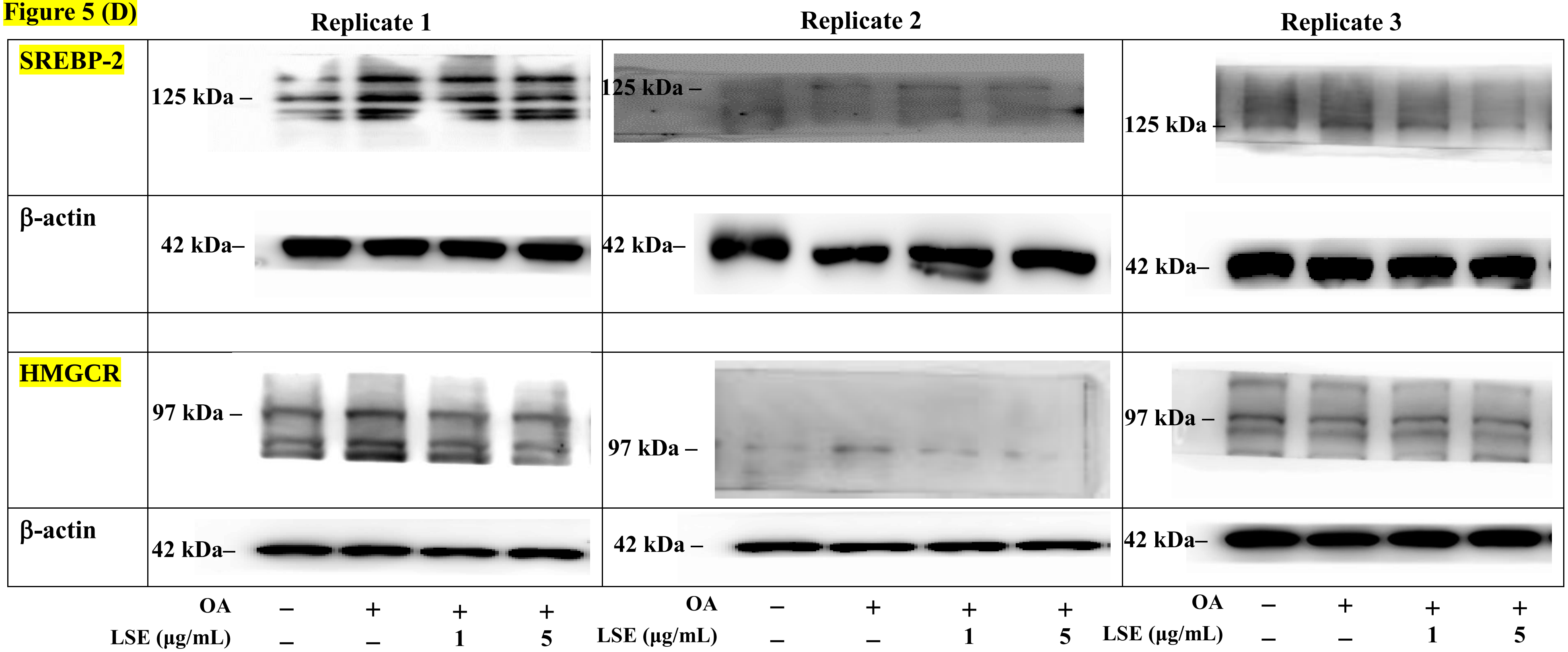
Replicate 1
Replicate 2
Replicate 3
SREBP-2
125 kDa –
β-actin
42 kDa–
HMGCR
97 kDa –
β-actin
42 kDa–
OA – + + +
LSE (μg/mL) – – 1 5

Figure 5 (E)

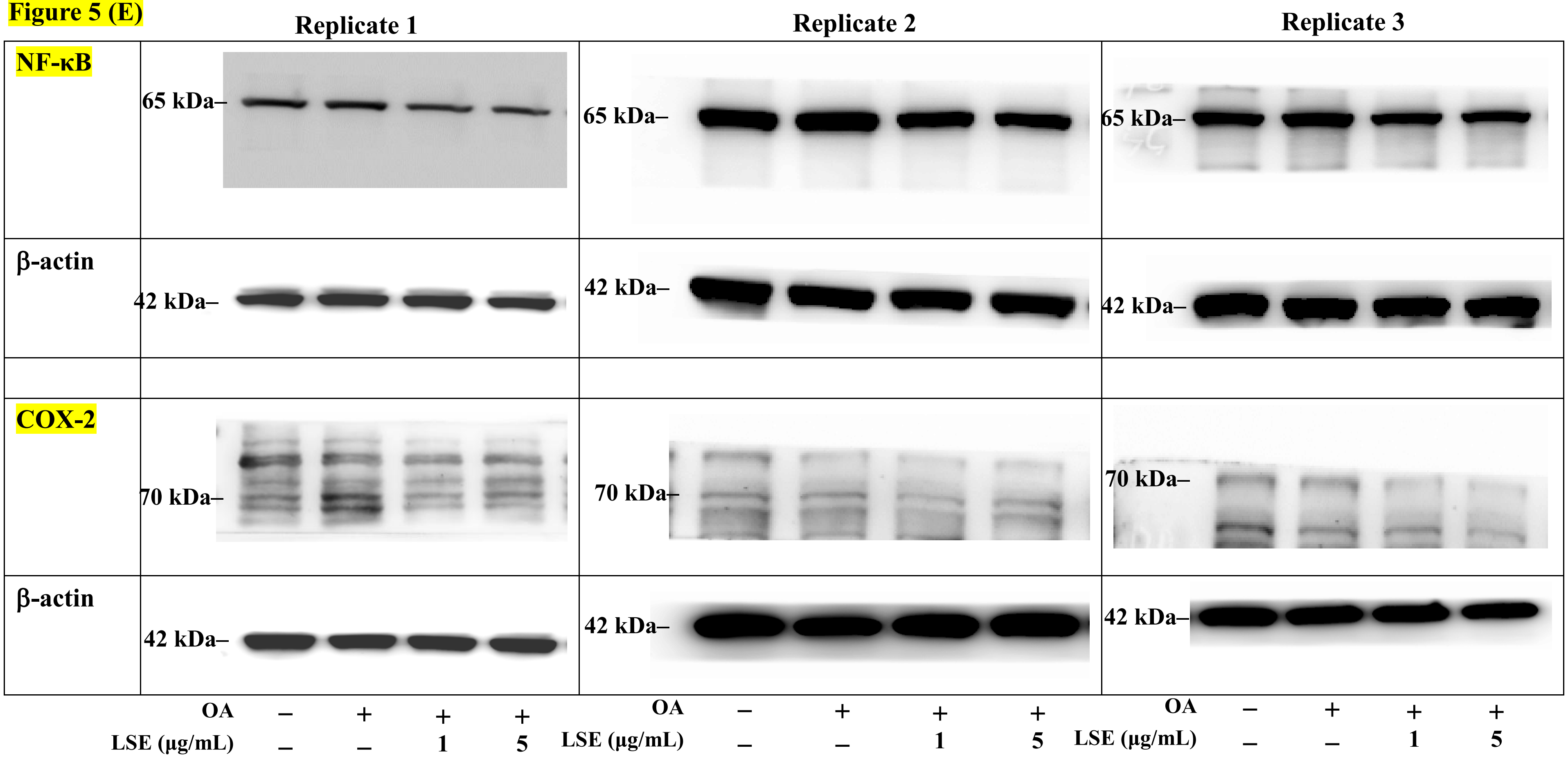
Replicate 1
Replicate 2
Replicate 3
NF-κB
65 kDa–
β-actin
42 kDa–
COX-2
70 kDa–
β-actin
42 kDa–
OA – + + +
LSE (μg/mL) – – 1 5

Figure 6 (D)

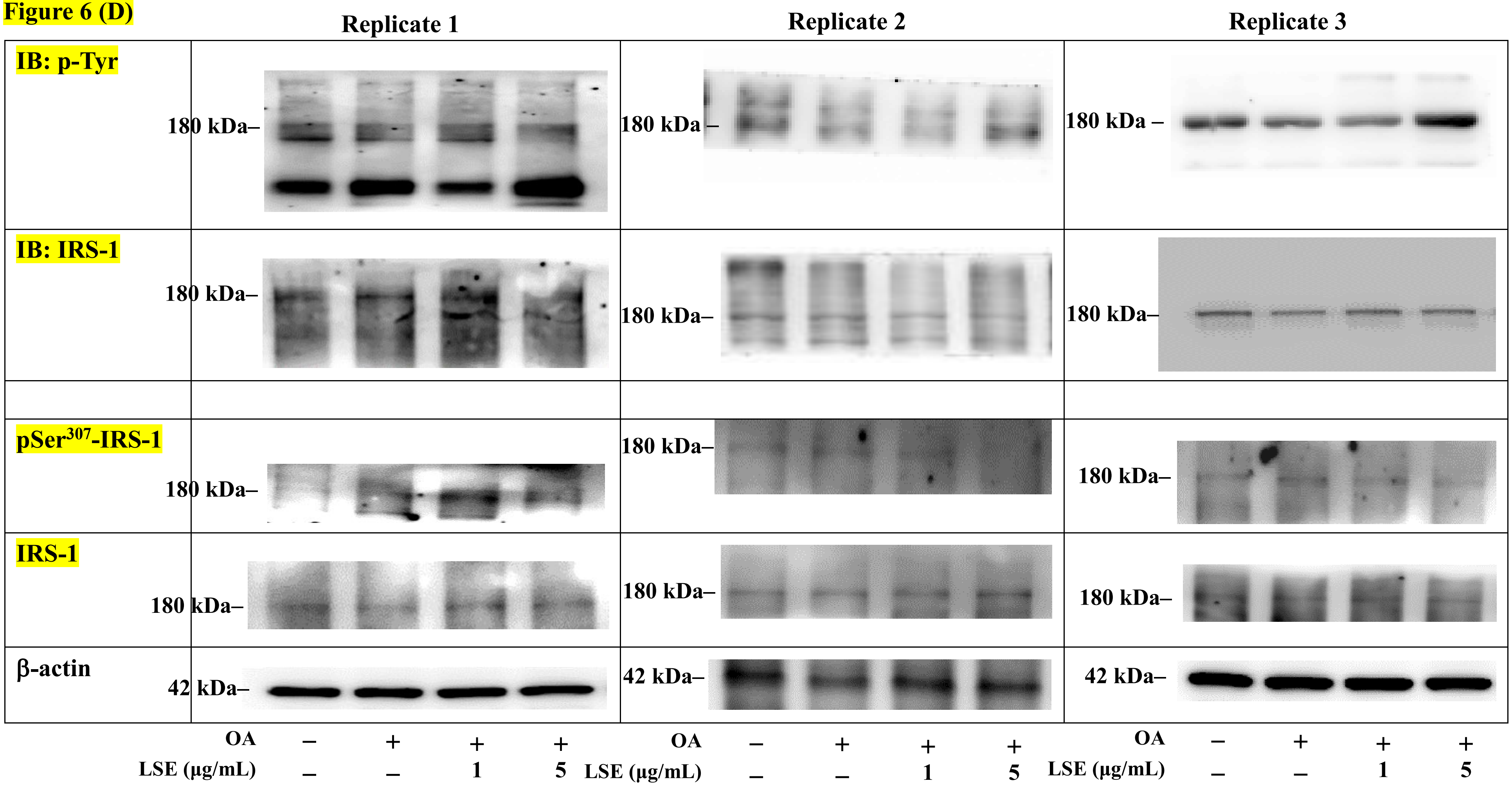

Replicate 1
Replicate 2
Replicate 3
IB: p-Tyr
IB: IRS-1
pSer307-IRS-1
IRS-1
β-actin
180 kDa–
42 kDa–
OA – + + +
LSE (μg/mL) – – 1 5

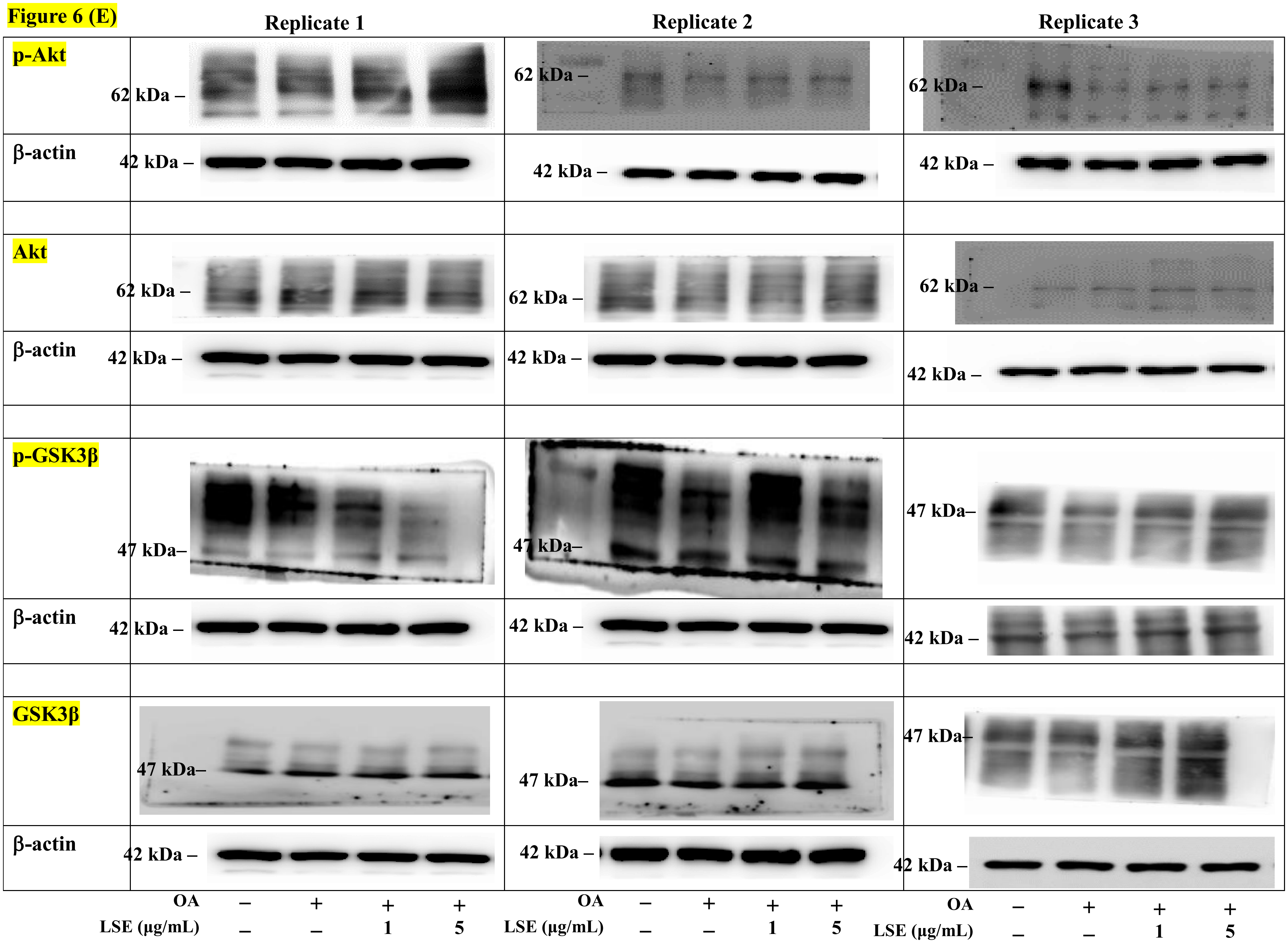
Figure 6 (E)
Replicate 1
Replicate 2
Replicate 3
p-Akt
62 kDa –
β-actin
42 kDa –
Akt
62 kDa –
β-actin
42 kDa –
p-GSK3β
47 kDa–
β-actin
42 kDa –
GSK3β
47 kDa–
β-actin
42 kDa –
OA – + + +
LSE (μg/mL) – – 1 5

Figure 6 (F)

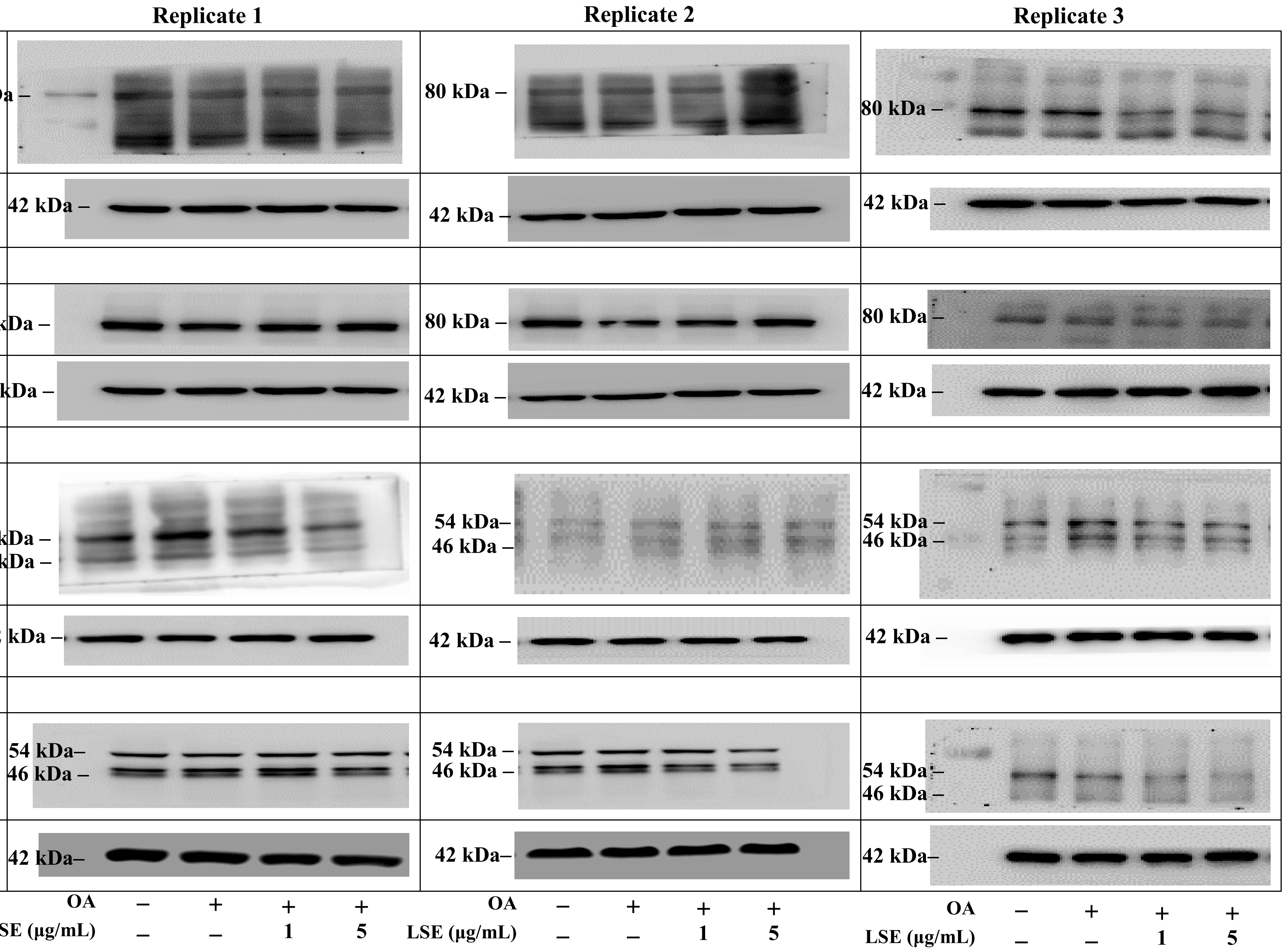
Replicate 1
Replicate 2
Replicate 3
p-PKC
80 kDa –
β-actin
42 kDa –
PKC
80 kDa –
β-actin
42 kDa –
p-JNK1/2
54 kDa –
46 kDa –
β-actin
42 kDa –
JNK
54 kDa –
46 kDa –
β-actin
42 kDa –
OA – + + +
LSE (μg/mL) – – 1 5

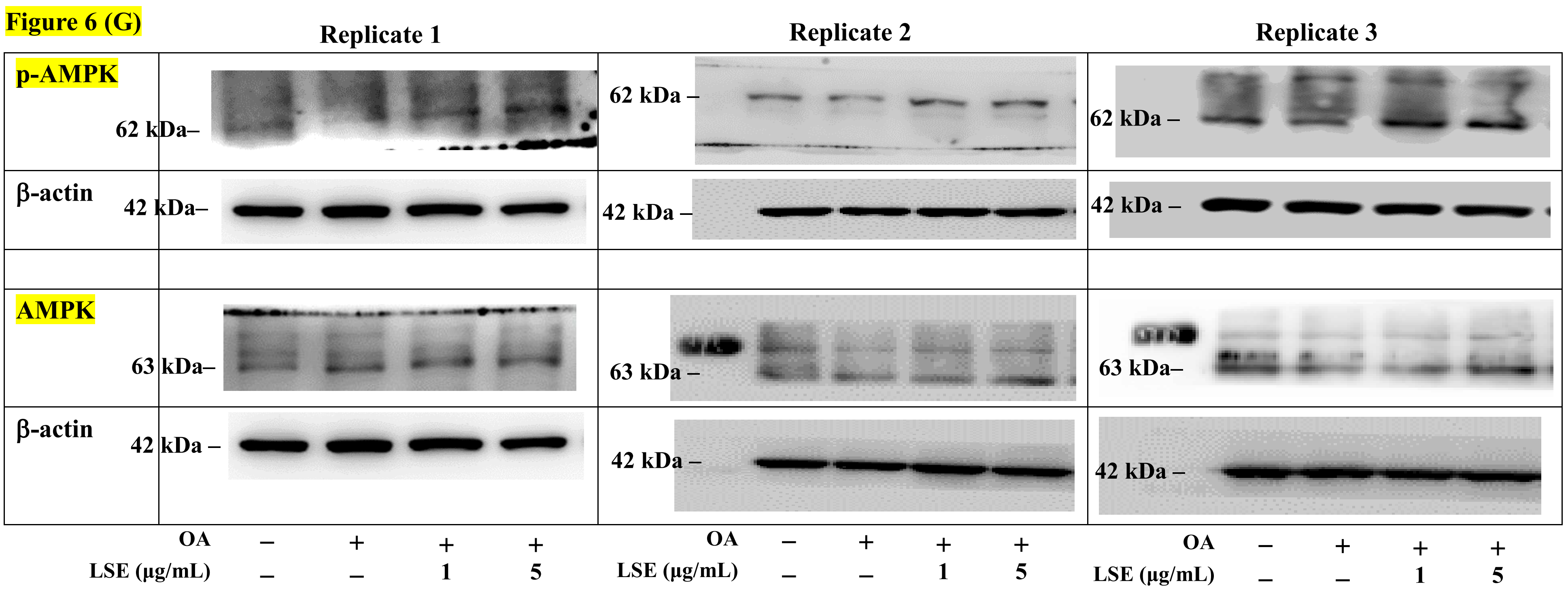

Figure 6 (G)
Replicate 1
Replicate 2
Replicate 3
p-AMPK
62 kDa–
62 kDa –
62 kDa –
β-actin
42 kDa–
42 kDa –
42 kDa –
AMPK
63 kDa–
63 kDa –
63 kDa–
β-actin
42 kDa –
42 kDa –
42 kDa –
OA – + + +
LSE (µg/mL) – – 1 5
OA – + + +
LSE (µg/mL) – – 1 5
OA – + + +
LSE (µg/mL) – – 1 5

Figure 7 (A)

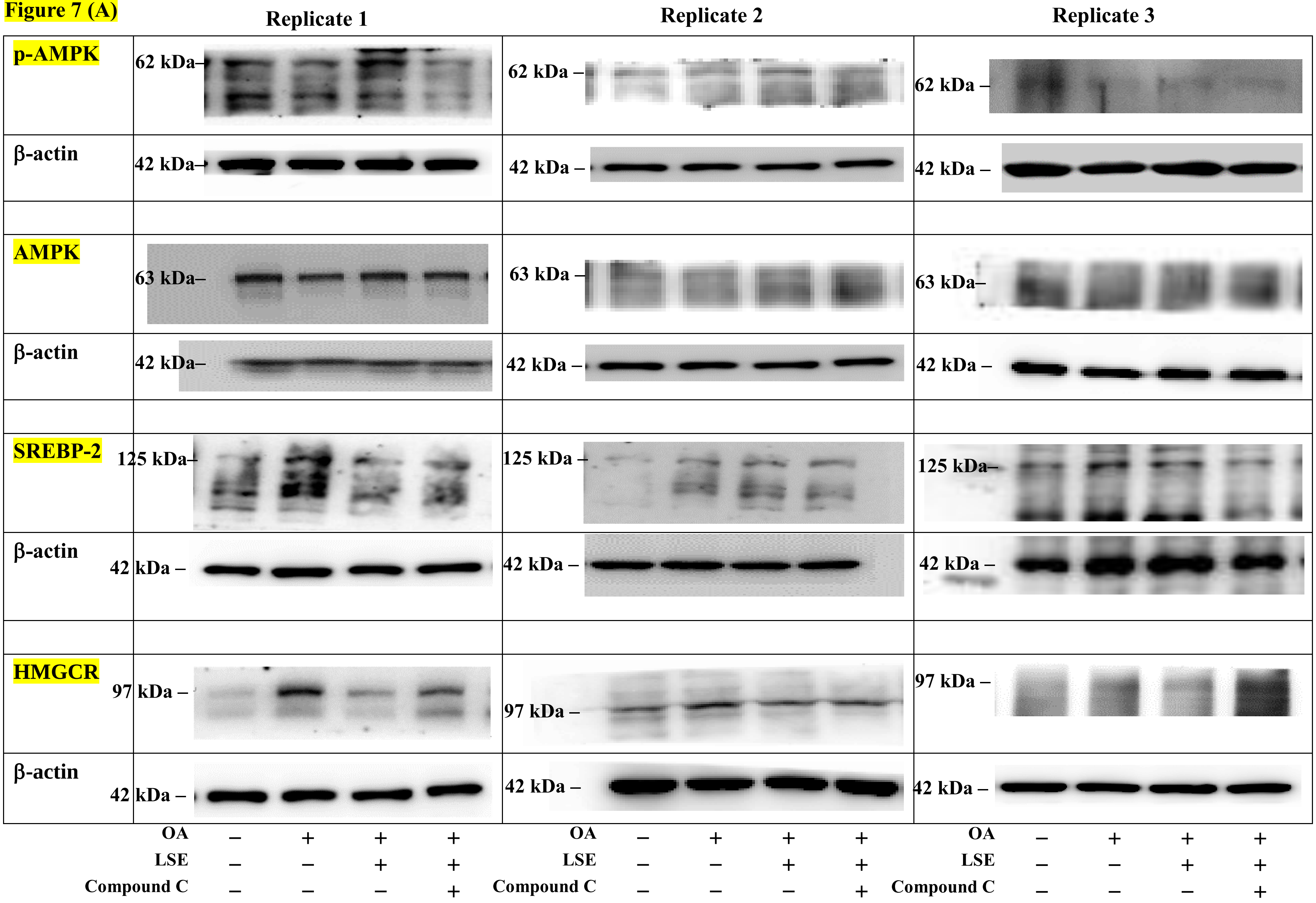
Replicate 1
Replicate 2
Replicate 3
p-AMPK
62 kDa
β-actin
42 kDa
AMPK
63 kDa
SREBP-2
125 kDa
HMGCR
97 kDa
OA – + + +
LSE – – + +
Compound C – – – +

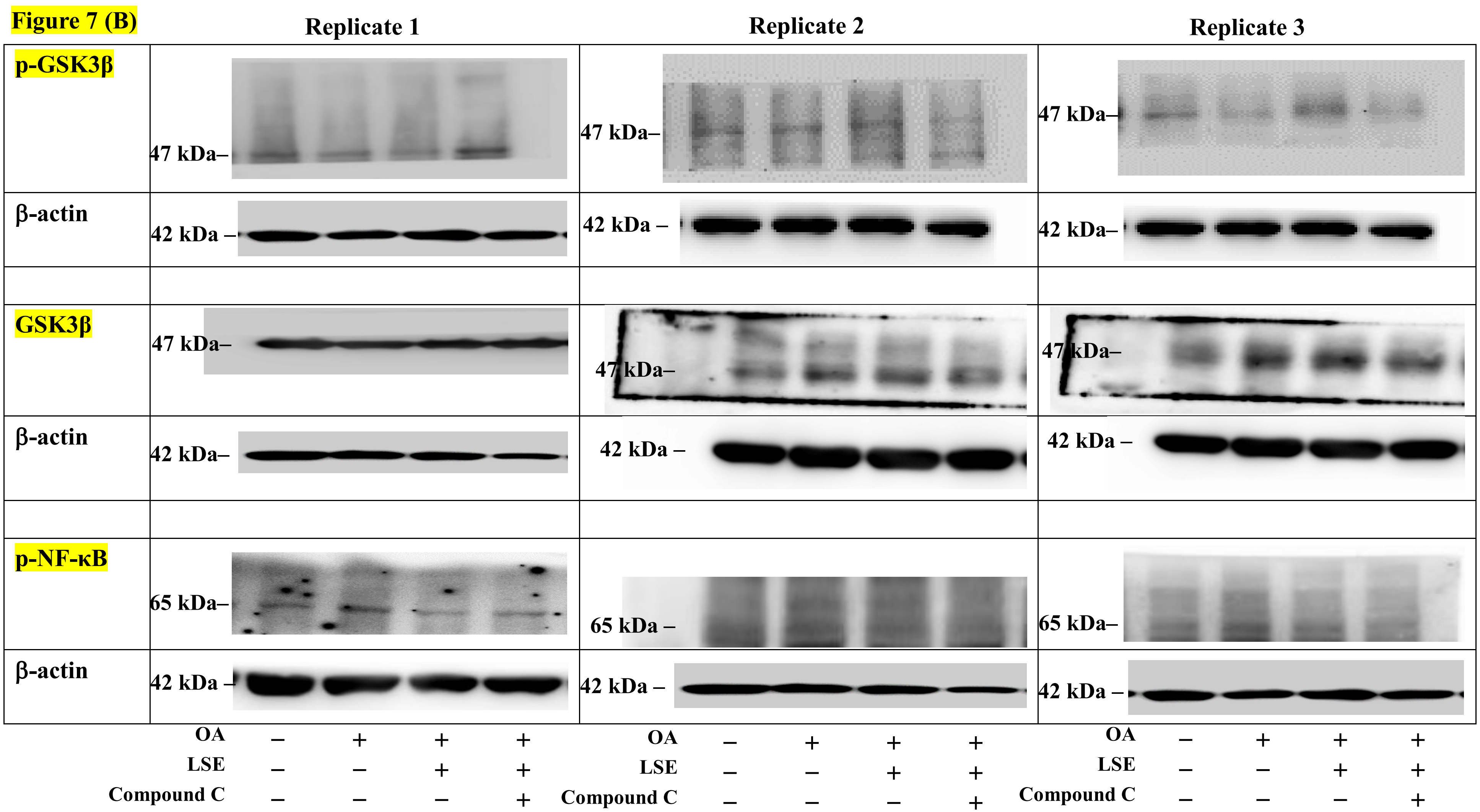

Figure 7 (B)
Replicate 1
Replicate 2
Replicate 3
p-GSK3β
47 kDa–
β-actin
42 kDa –
GSK3β
47 kDa–
β-actin
42 kDa–
p-NF-κB
65 kDa–
β-actin
42 kDa –
OA – + + +
LSE – – + +
Compound C – – – +

Figure 7 (B). (continued)

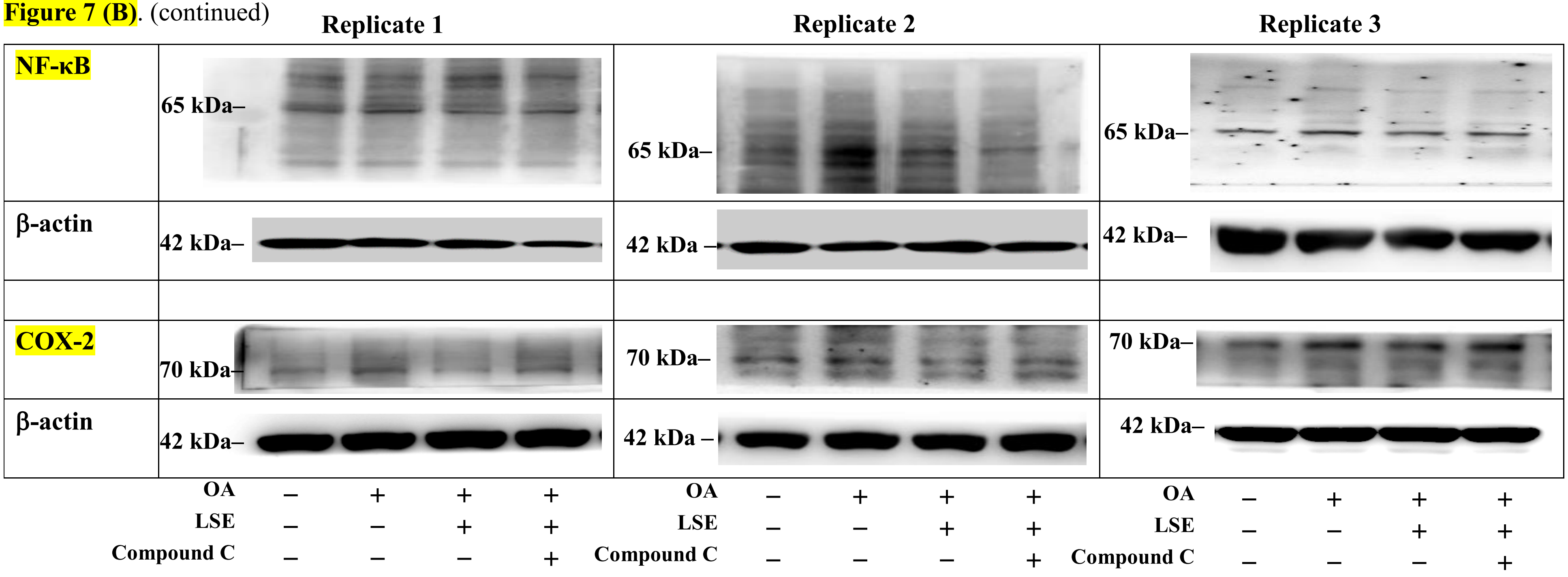

Figure S3. The original Western blot of Figures 4A-D, 5D, 5E, 6D-G, and 7A-B.

Representative images from three independent biological replicates (n = 3) show original blots with molecular weight markers. Each lane is annotated to indicate the corresponding experimental group.

Figure S4 (A)

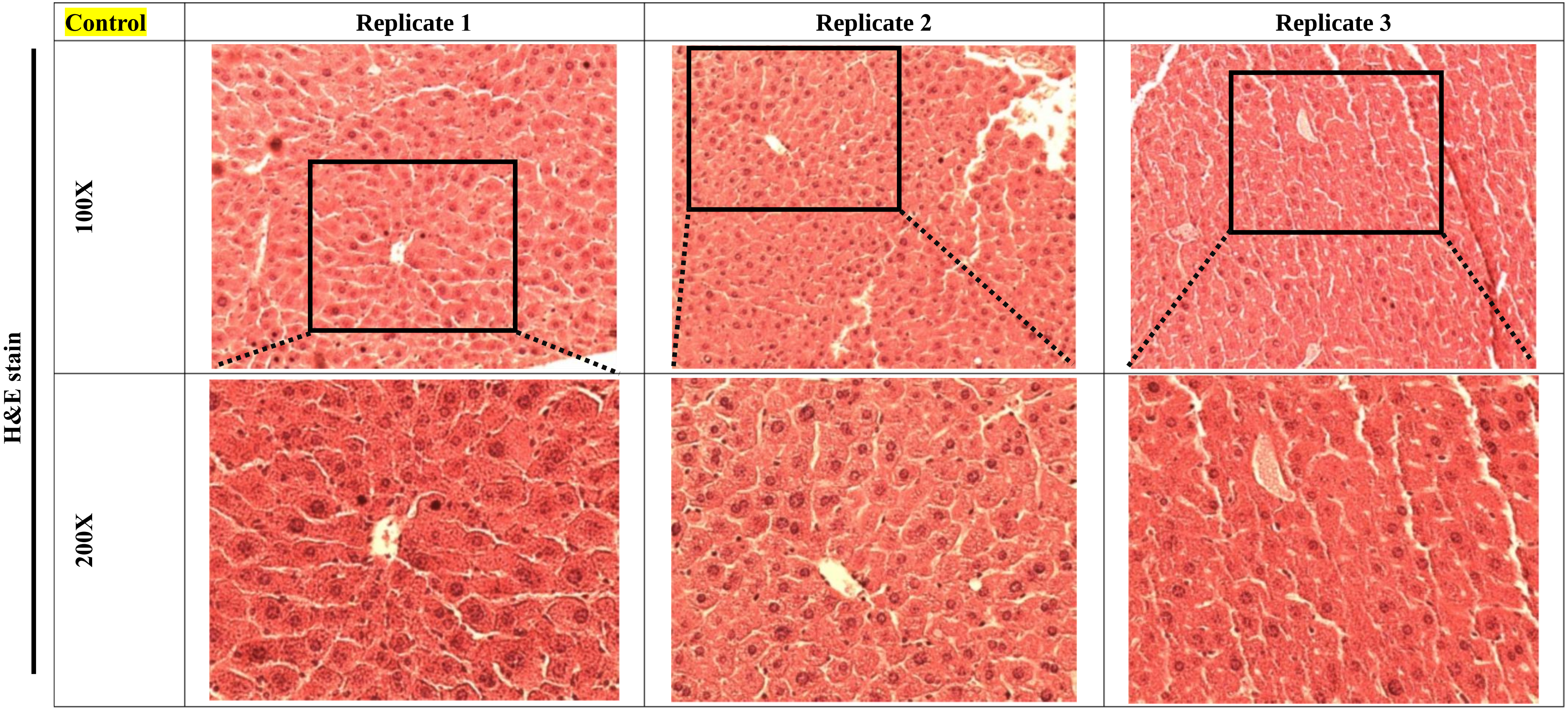
Control
Replicate 1
Replicate 2
Replicate 3
H&E stain
100X
200X

Figure S4 (A). (continued)

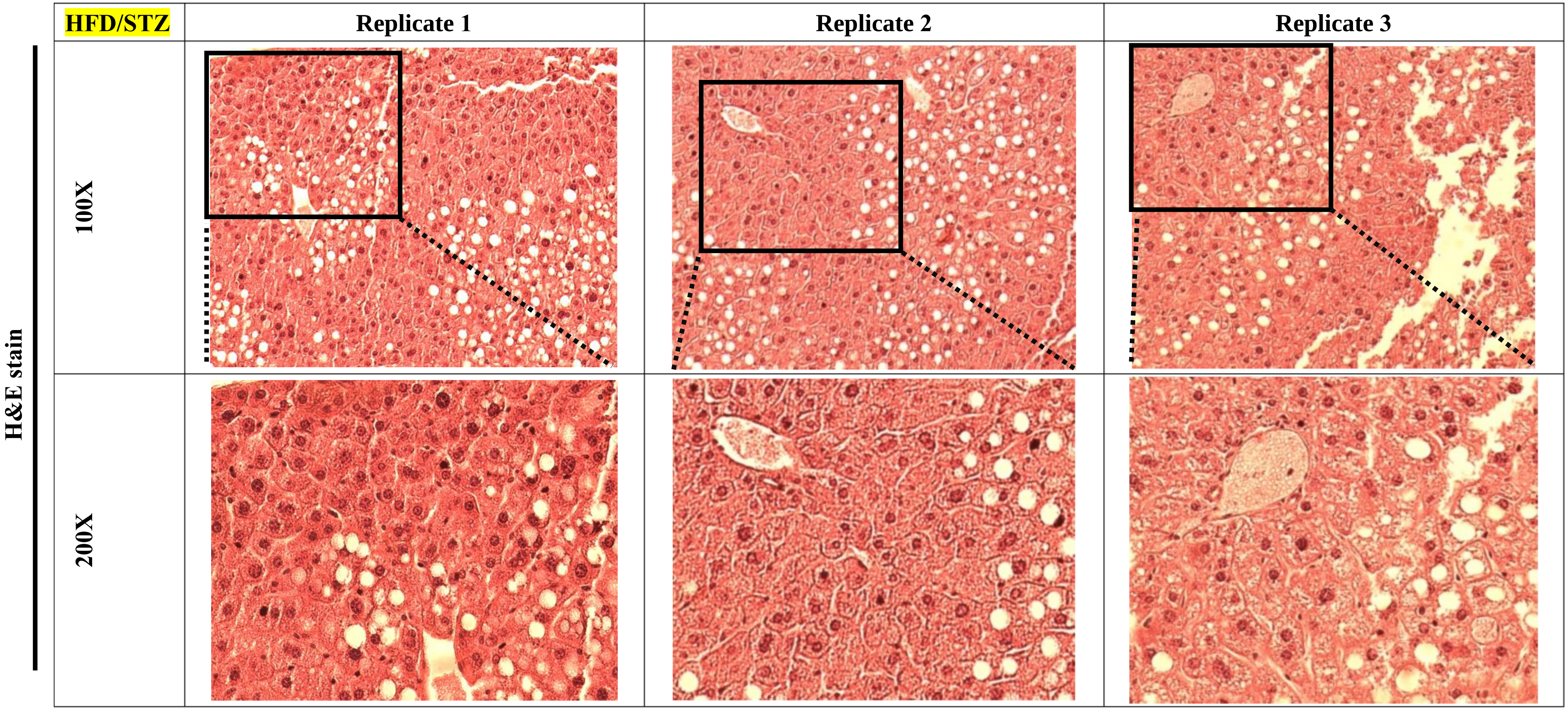

Figure S4 (A). (continued)

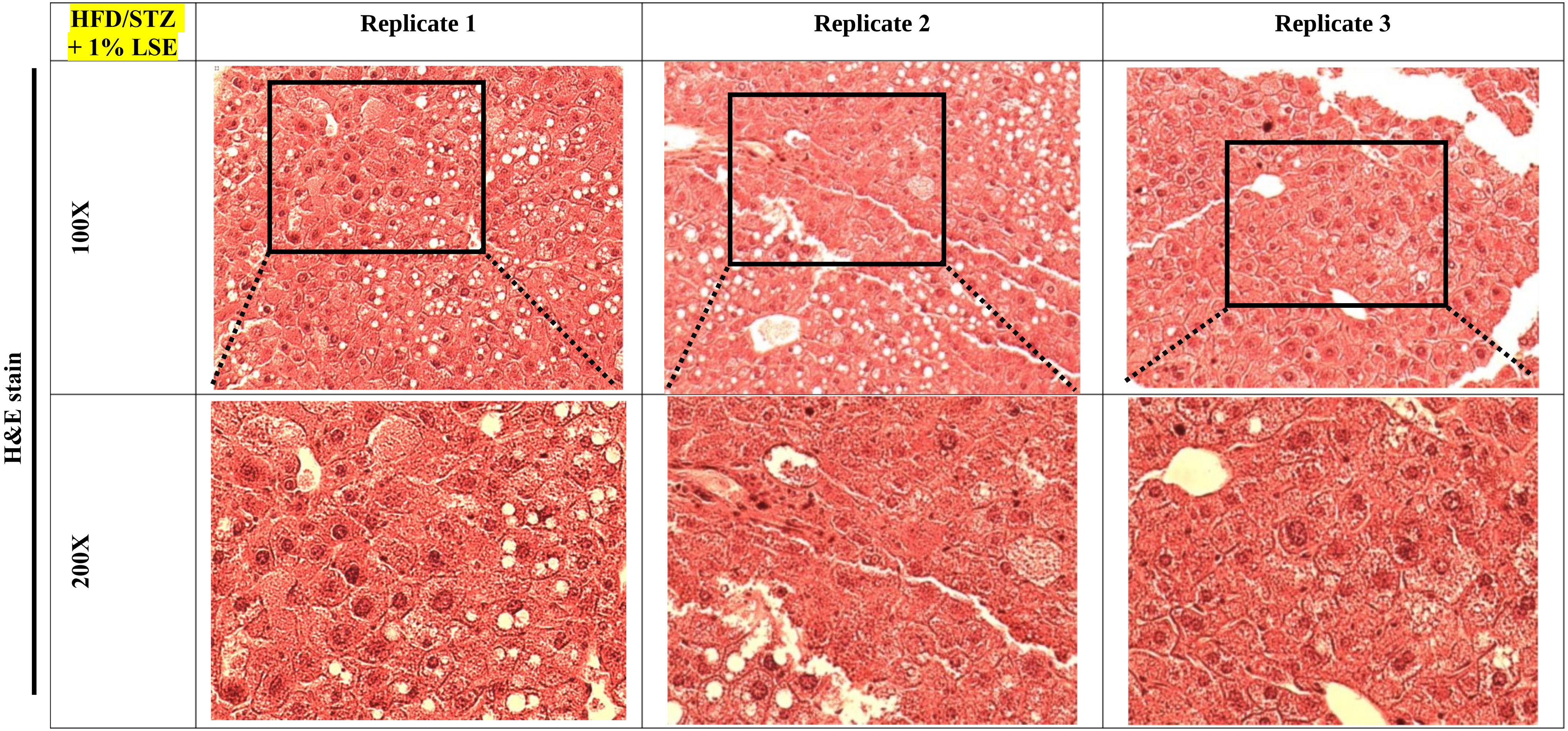

Figure S4 (A). (continued)

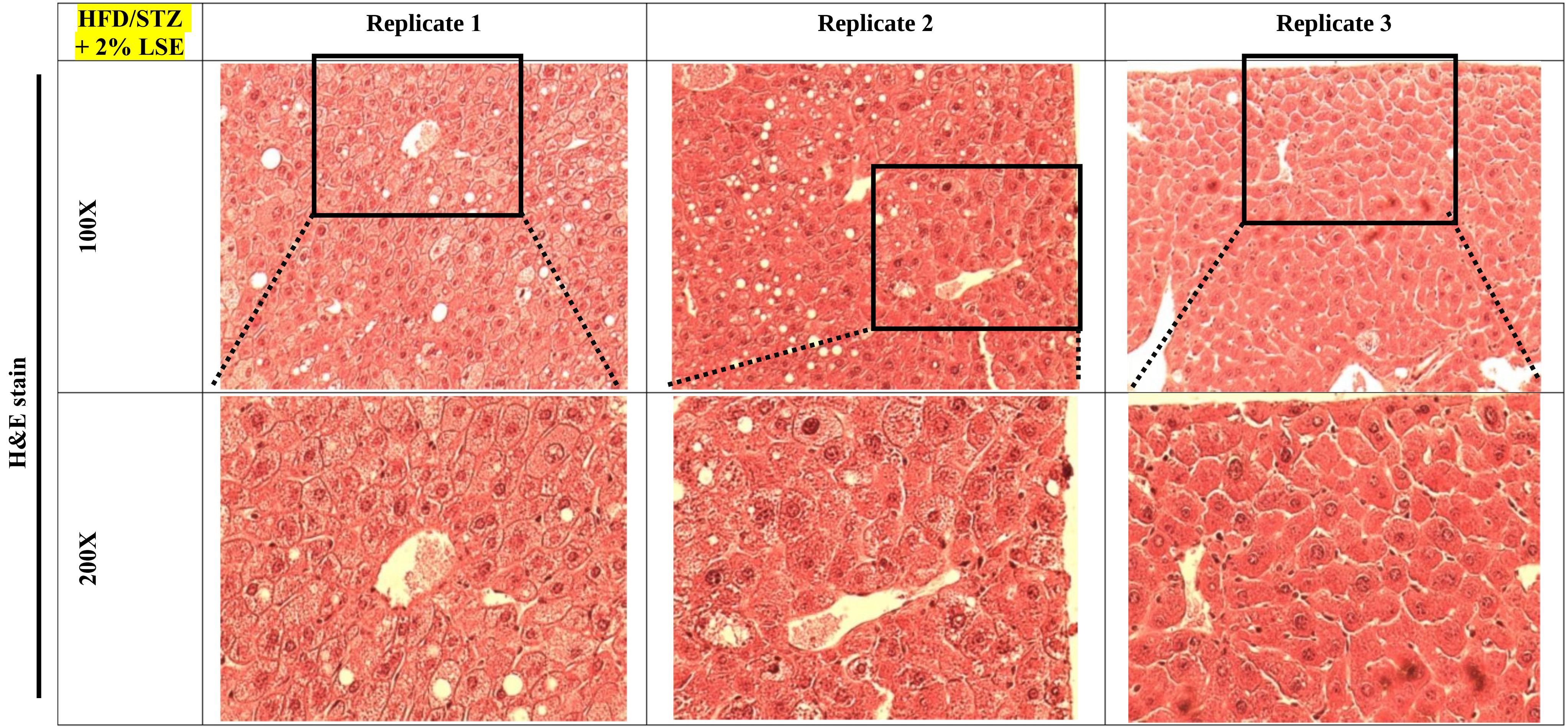

Figure S4 (A). (continued)

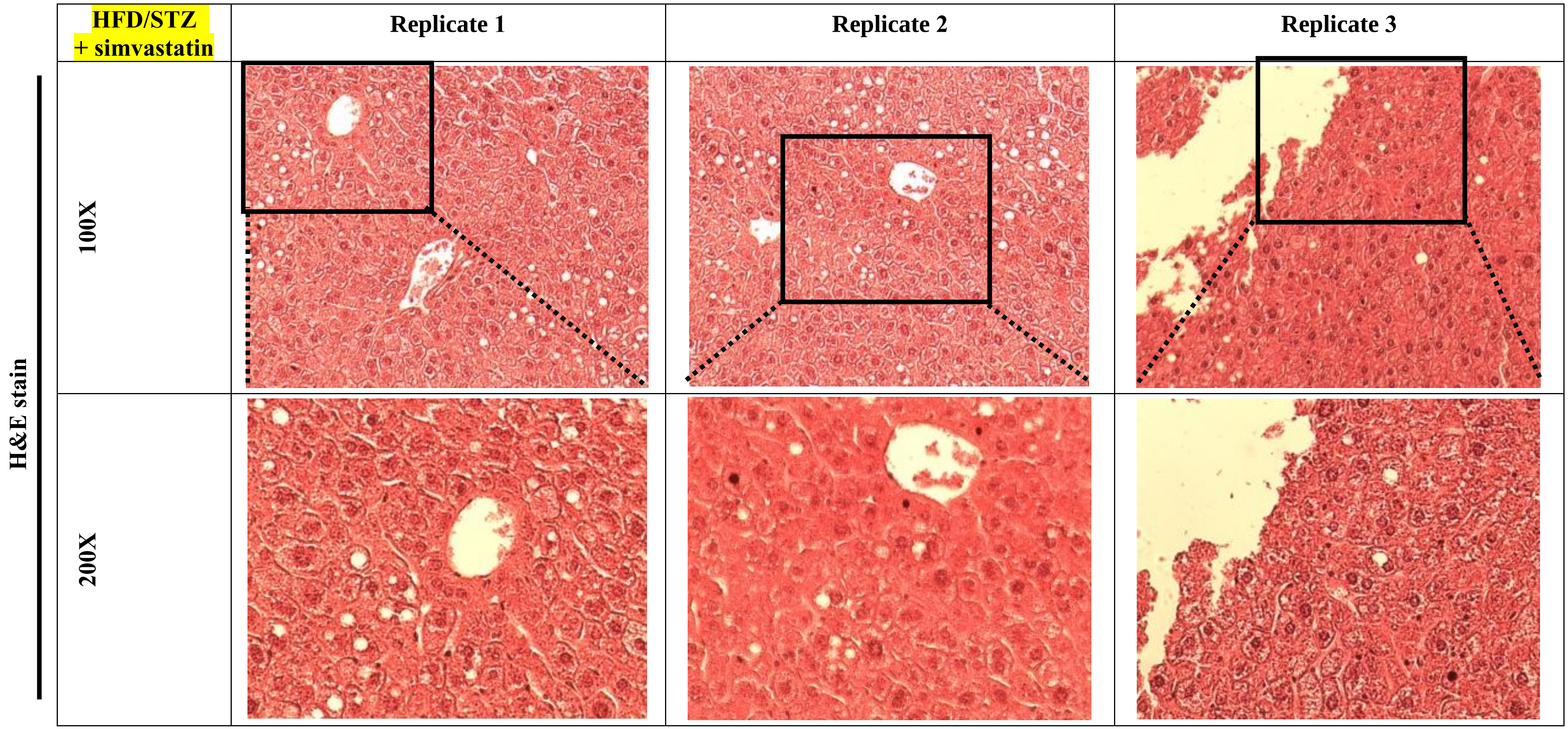

Figure S4 (B)

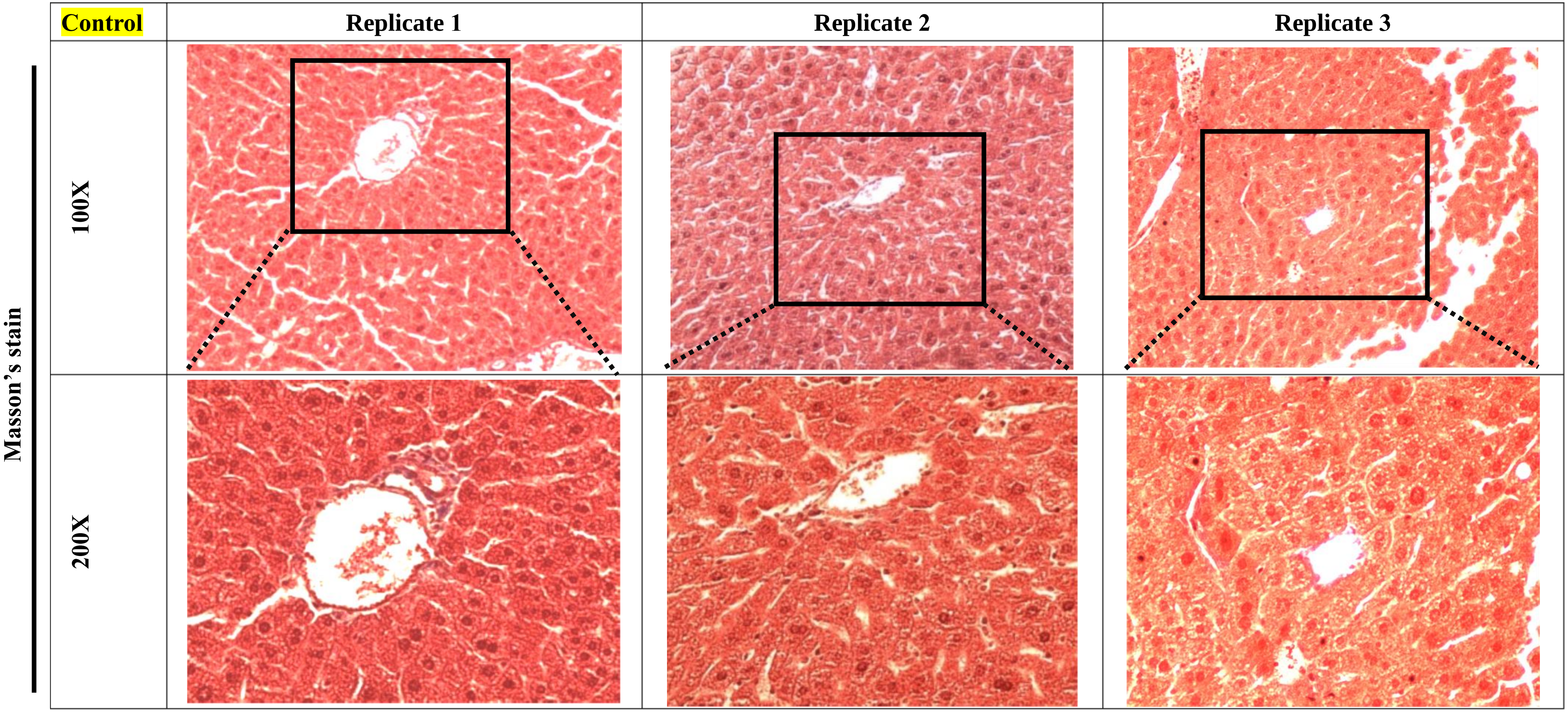
Control
Replicate 1
Replicate 2
Replicate 3
Masson's stain
100X
200X

Figure S4 (B). (continued)

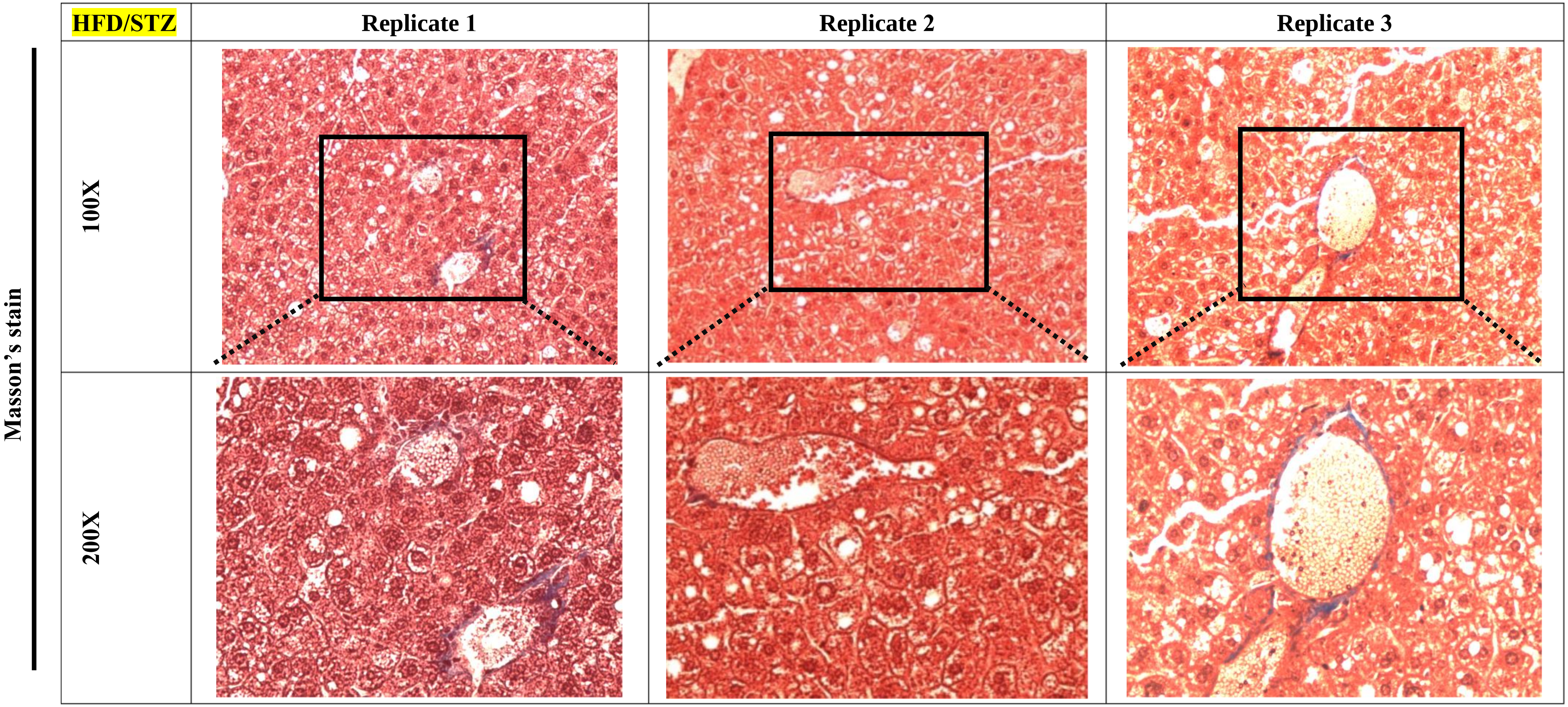

Figure S4 (B). (continued)

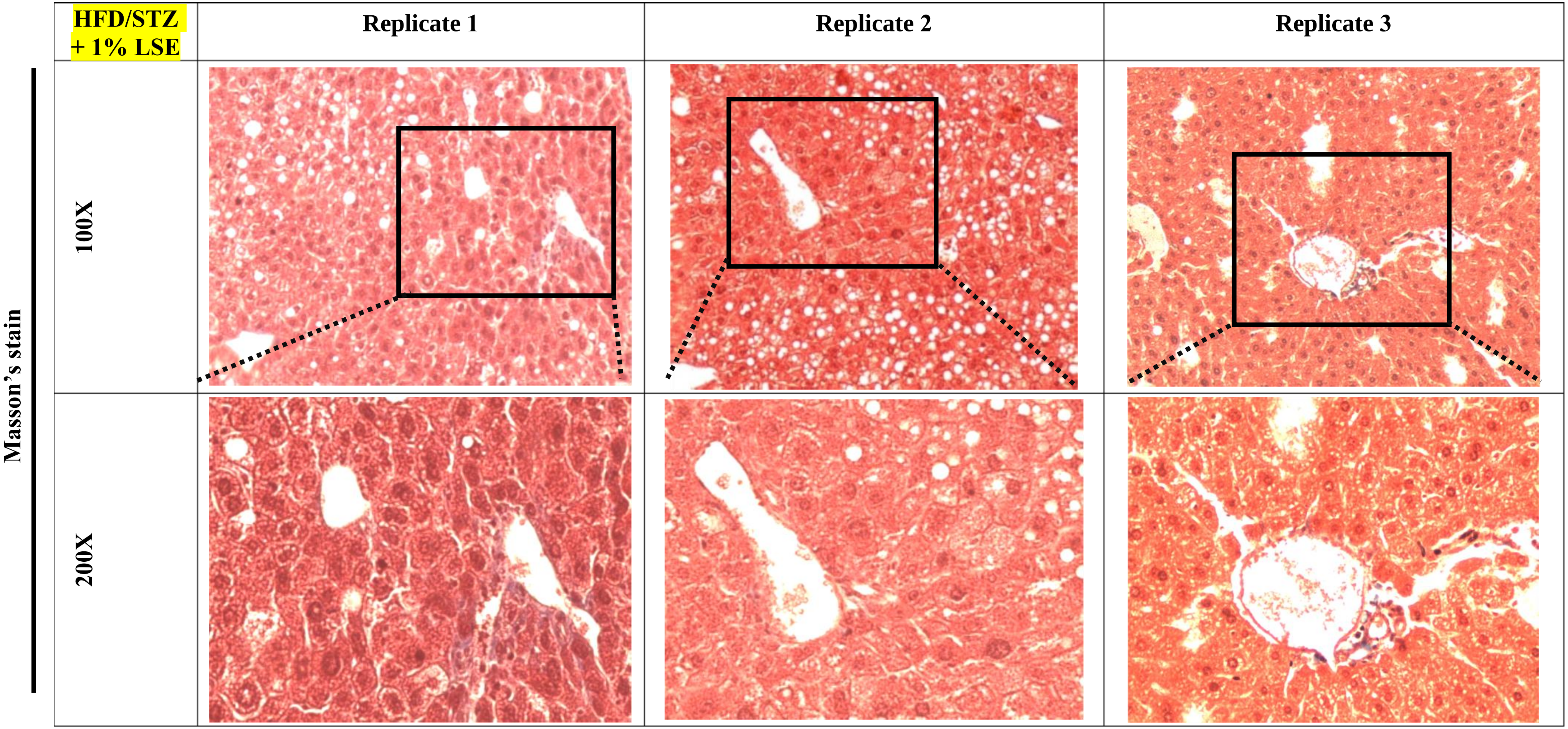

Figure S4 (B). (continued)

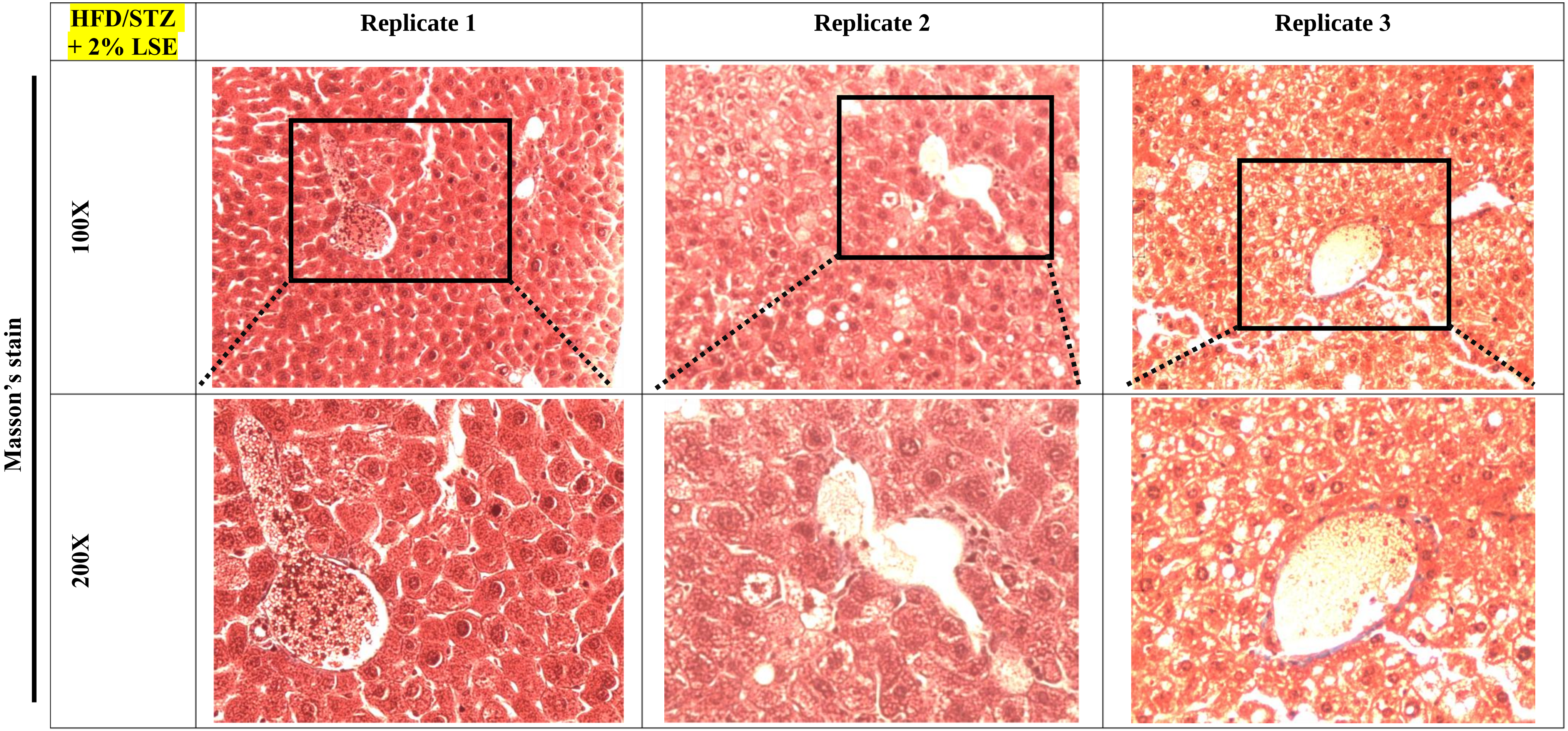

Figure S4 (B). (continued)

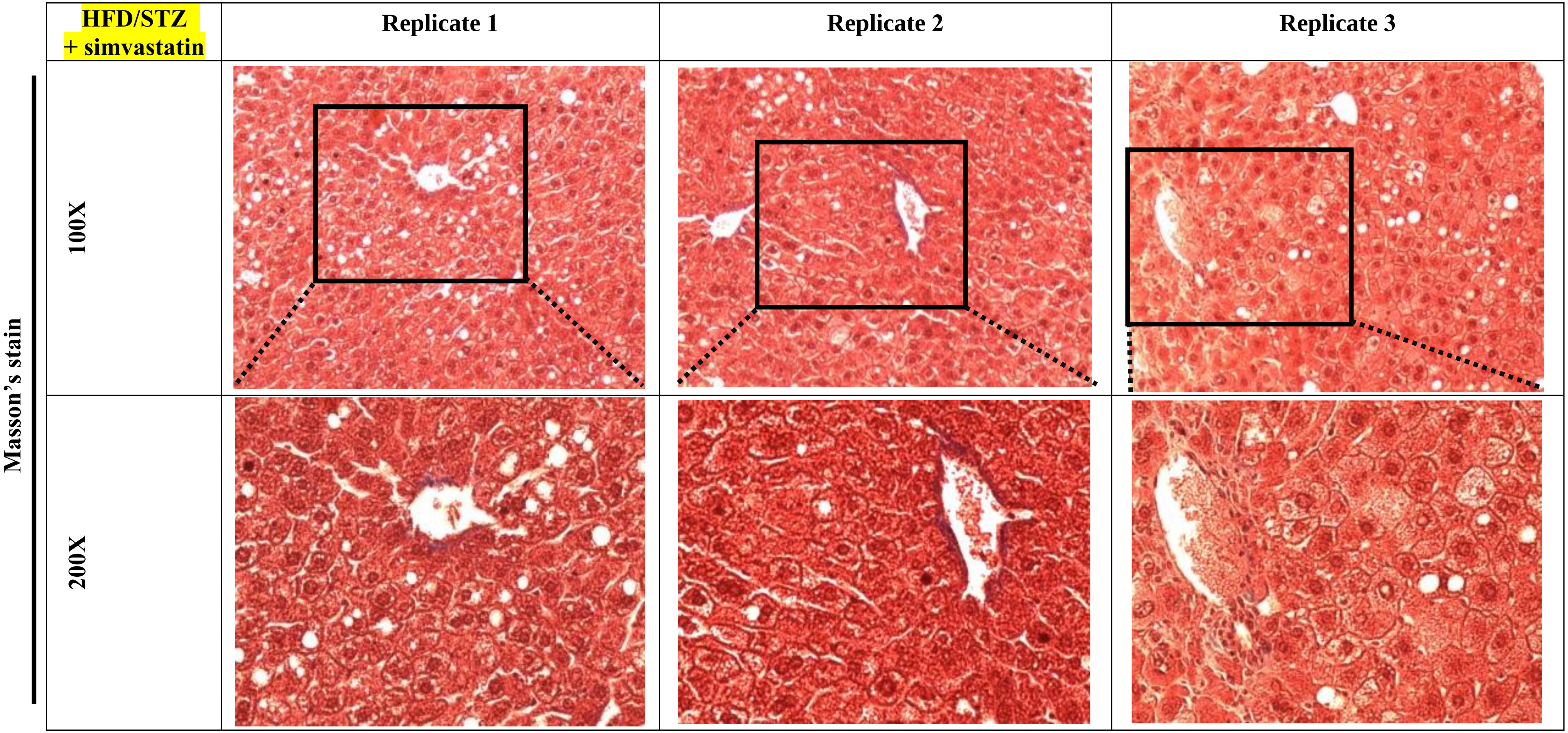

Figure S4. The original H&E stain of Figure 3A and Masson's stain of Figure 3B.

Representative images show results from three independent biological replicates (magnification, x100, *top panels*; and x200, *bottom panels*).
